# CDK12 regulates cellular metabolism to promote glioblastoma growth

**DOI:** 10.1172/jci.insight.190780

**Published:** 2025-09-25

**Authors:** Jeong-Yeon Mun, Chang Shu, Qiuqiang Gao, Zhe Zhu, Hasan O. Akman, Mike-Andrew Westhoff, Georg Karpel-Massler, Markus D. Siegelin

**Affiliations:** 1 Department of Pathology and Cell Biology, and; 2 Department of Neurology, Columbia University Medical Center, New York, New York, USA.; 3 Department of Pediatrics and Adolescent Medicine, and; 4 Department of Neurosurgery, Ulm University Medical Center, Ulm, Germany.

**Keywords:** Metabolism, Oncology, Apoptosis, Brain cancer, Oncogenes

## Abstract

Glioblastoma IDH-wildtype is the most common and aggressive primary brain tumor in adults, with poor prognosis despite current therapies. To identify new therapeutic vulnerabilities, we investigated the role of CDK12, a transcription-associated cyclin-dependent kinase, in glioblastoma. Genetic or pharmacologic inactivation of CDK12 impaired tumor growth in patient-derived xenograft (PDX) models and enhanced the efficacy of temozolomide. Metabolic profiling using extracellular flux analysis and stable isotope tracing with U-¹³C-glucose and U-¹³C-glutamine showed that CDK12 inhibition disrupted mitochondrial respiration, resulting in energy depletion and apoptotic cell death characterized by caspase activation and Noxa induction. Mechanistically, we identified a direct interaction between CDK12 and GSK3β. CDK12 inhibition activated GSK3β, leading to downregulation of PPARD, a transcriptional regulator of oxidative metabolism. This CDK12/GSK3β/PPARD axis was required for glioblastoma cell proliferation and metabolic homeostasis. In vivo, CDK12 inhibition significantly extended survival without overt toxicity and induced complete tumor regression in a subset of animals. Strikingly, combined CDK12 inhibition and temozolomide treatment led to complete tumor eradication in all animals tested. These findings establish CDK12 as a key regulator of glioblastoma metabolism and survival, and provide strong preclinical rationale for its therapeutic targeting in combination with standard-of-care treatments.

## Abstract 2

Targeting the protein CDK12 is a promising therapeutic approach for glioblastoma. This approach significantly prolongs survival and can completely eliminate tumors when combined with standard treatment in mouse models.

## Introduction

Glioblastoma (GBM) IDH-wildtype, classified as WHO grade IV and characterized by wild-type IDH1, has now emerged as a distinct entity, significantly streamlining research and guiding the development of novel therapies (1–5). The latest version of the WHO classification has distinguished IDH1-mutant GBMs as a distinct diagnosis called astrocytoma WHO grade IV, IDH1 mutant. This new categorization scheme aims to streamline both diagnosis and treatment processes for these tumors. GBMs with a wild-type IDH1 typically have a poor prognosis of 1 to 1.5 years from the time of diagnosis despite aggressive treatments such as surgical resection followed by radiation and temozolomide (TMZ) administration (6). Clinical trials have indicated that tumor treatment fields may offer a slight increase in survival for patients with GBM, highlighting the urgent need for improved treatments specific to wild-type IDH1 GBMs (7). When it comes to transcriptional subtypes, wild-type IDH1 GBMs can be categorized into 4 major categories, with 3 being the most commonly used: proneural, classical, and mesenchymal. It is worth noting that mesenchymal GBMs form a category of their own but also arise from the proneural subtype after treatment and resistance formation (8).

Energy metabolism primarily involves glycolysis and cellular respiration linked to oxidative phosphorylation. Although tumor cells are recognized for generating high levels of lactate in the Warburg effect, the oxidation of energy-dense substances in the tricarboxylic acid (TCA) cycle and the respiratory transport chain remains crucial for GBM and other solid malignancies, particularly in the outer regions of such tumors where even lactate can be oxidized to generate ATP (9–14). Therefore, comprehending how oxidative energy metabolism is regulated is essential, as GBM relies on this pathway for its growth. Both glycolytic and oxidative metabolism are regulated by transcriptional regulation, involving mediators out of the PPAR family of transcription factors, including PGC1A, PGC1B, and PPARD (15–17). Previous studies from our group have illuminated the role of PGC1A in conferring resistance to histone deacetylase inhibitor therapies across multiple GBM model systems. These findings suggest that PGC1A promotes metabolic reprogramming, specifically through the enhancement of fatty acid oxidation, in GBM cells (18). The role of PGC1A has been more extensively characterized in cancer model systems (19–24), whereas the function of PPARD remains comparatively less understood (25).

Glycogen synthase kinase 3β (GSK3β) has been implicated in the regulation of cell death and proliferation in GBM. Notably, GSK3β plays a key role in modulating the activity of the critical transcription factor, c-Myc. When GSK3β is active, it promotes the proteasomal degradation of c-Myc (26). This connection has been a subject of extensive study in GBM, as the stem-like GBM cells, which are considered drivers of disease recurrence, exhibit a high dependence on c-Myc for maintaining their undifferentiated state (27–30).

Cyclin-dependent kinases (CDKs) play a crucial role in regulating the cell cycle and influencing transcription in various types of cancer, including GBM. Several CDKs, such as CDK4, CDK7, CDK9, and more recently CDK12, have been closely studied for their involvement in cancer progression. In estrogen receptor–positive breast cancer specifically, CDK4 has become a specific target; its activity is inhibited by palbociclib to suppress breast cancer growth both in vitro and in mouse models as well as among patients with breast cancer (31). Attempts have been made to target CDK4 in GBM, but with limited success. CDK7 has been recognized as a promising target for treating different forms of both solid and non-solid cancers (32, 33). This is because this enzyme plays a crucial role in transcription by adding phosphate groups to RNA polymerase II. Furthermore, CDK7 contributes significantly to the control of clustered enhancer regions referred to as super-enhancers, along with various other components including histone deacetylases. Targeting CDK7 has been significantly hindered by the fact that the most abundantly used inhibitor, THZ1, is not only specific against CDK7 but also substantially blocks the activity of CDK12 and CDK13 (34–37). While these features may be beneficial given that all 3 kinases are relevant for cancer development and progression, THZ1 is not well tolerated in vivo and has unfavorable pharmacokinetics (34–37). More precise and better-tolerated inhibitors targeting CDK7 and CDK12 are necessary in order to gain a complete understanding of the potential of these kinases as therapeutic targets in GBM, as well as other types of cancer.

CDK12 has not received much attention as a potential cancer treatment, except in cases like triple-receptor negative breast cancer model systems. Its interaction with cyclin-K affects transcription similarly to CDK7, and previous research indicates its role in DNA repair. Loss of CDK12 function or treatment with a novel selective CDK12 inhibitor disrupts this interaction, consequently blocking CDK12 activity (38–42). However, the exploration of CDK12 as a major therapeutic target for GBM remains almost completely uncharted territory.

This study identifies CDK12 as a key driver of GBM growth and resistance to both cell death and standard therapy. Unexpectedly, we found that CDK12 also regulates energy metabolism in GBM, partly through 2 interconnected intermediaries. CDK12 directly interacts with and regulates GSK3β, which in turn influences tumor metabolism and growth. PPARD, a downstream target of GSK3β and a modulator of oxidative metabolism, emerged as a mediator of therapy resistance. Strikingly, CDK12 loss eliminated tumors in some orthotopic patient-derived xenograft (PDX) GBM models, and its combination with TMZ eradicated tumors in all treated animals. These findings establish CDK12 as a promising therapeutic target in GBM.

## Results

### Inhibition of CDK12 enhances apoptosis and reduces viability in GBM.

**Figure 1:**
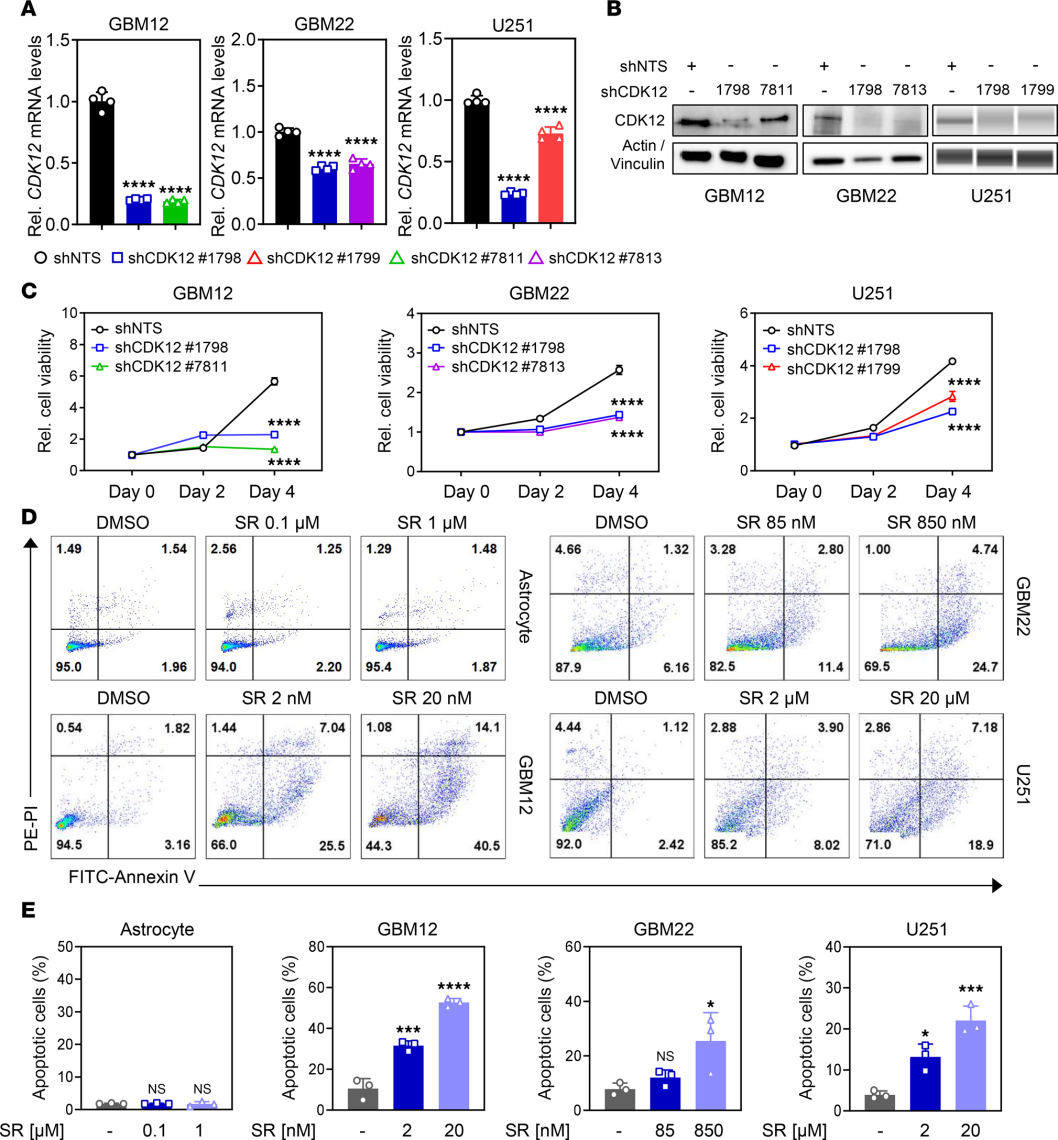
Inhibition of CDK12 in GBM cells reduces cell proliferation and increases cell apoptosis. (**A**) The qPCR results and (**B**) Western blot results show analysis of CDK12 expression in the indicated 4 types of shCDK12 stable knockdown samples in multiple GBM cells. The qPCR data are presented as mean ± SD. (**C**) Stable shCDK12 cells or shNTS cells were assessed for cell viability for 0, 2, and 4 days. Data are presented as mean ± SD. (**D** and **E**) Astrocytes and multiple GBM cells were treated with SR-4835 for 72 hours and were analyzed by flow cytometry following staining with annexin V/PI. The bar graph shows the percentage of apoptotic cells and data are presented as mean ± SD. *P < 0.05; ***P < 0.001; ****P < 0.0001 by 1-way ANOVA with Dunnett’s multiple-comparison test.

CRISPR and short hairpin RNA (shRNA) screens identified CDK12 as a key driver of GBM growth. Kaplan-Meier analysis of The Cancer Genome Atlas (TCGA) GBM cohort demonstrated a trend toward reduced overall survival in patients with high CDK12 expression (median 11.2 months) compared with those with low expression (median 17.9 months). However, this difference did not reach statistical significance (HR = 0.61, 95% confidence interval: 0.32–1.17; log-rank *P* = 0.1296; Wilcoxon’s *P* = 0.2184; Supplemental Figure 1A; supplemental material available online with this article; https://doi.org/10.1172/jci.insight.190780DS1). Quantitative PCR (qPCR) analysis confirmed CDK12 upregulation (1.6- to 6.1-fold) across GBM cell lines relative to human astrocytes (Supplemental Figure 1B). CDK12 expression was significantly elevated in GBM tumors compared with normal brain tissue (Supplemental Figure 1C). To assess CDK12’s function, we generated GBM cell lines with stable shCDK12-mediated knockdown and confirmed reduced mRNA and protein levels (Figure 1, A and B). Cell viability assays showed significant reductions in shCDK12 cells by day 4 (Figure 1C). Annexin V/PI staining and flow cytometry revealed increased apoptosis in both shCDK12 cells and cells treated with the selective CDK12/13 inhibitor SR-4835, with SR-4835 also reducing CDK12 protein levels in GBM12 cells (Figure 1, D and E, and Supplemental Figure 1, D–G). The IC_50_ values of multiple GBM cell lines were determined based on cell viability assays (Supplemental Figure 1H). SR-4835 induced robust apoptosis in GBM cell lines, including PDX and neurosphere models, while sparing normal astrocytes. GBM12 cells were especially sensitive, showing apoptosis at concentrations as low as 2 nM (Figure 1, D and E, and Supplemental Figure 1, E and F). Additionally, CDK13 was also upregulated in GBM, and its knockdown impaired cell viability by day 4 (Supplemental Figure 1, I and J).

### CDK12 regulates the metabolic transcriptome, with strong effects on oxidative phosphorylation.

**Figure 2:**
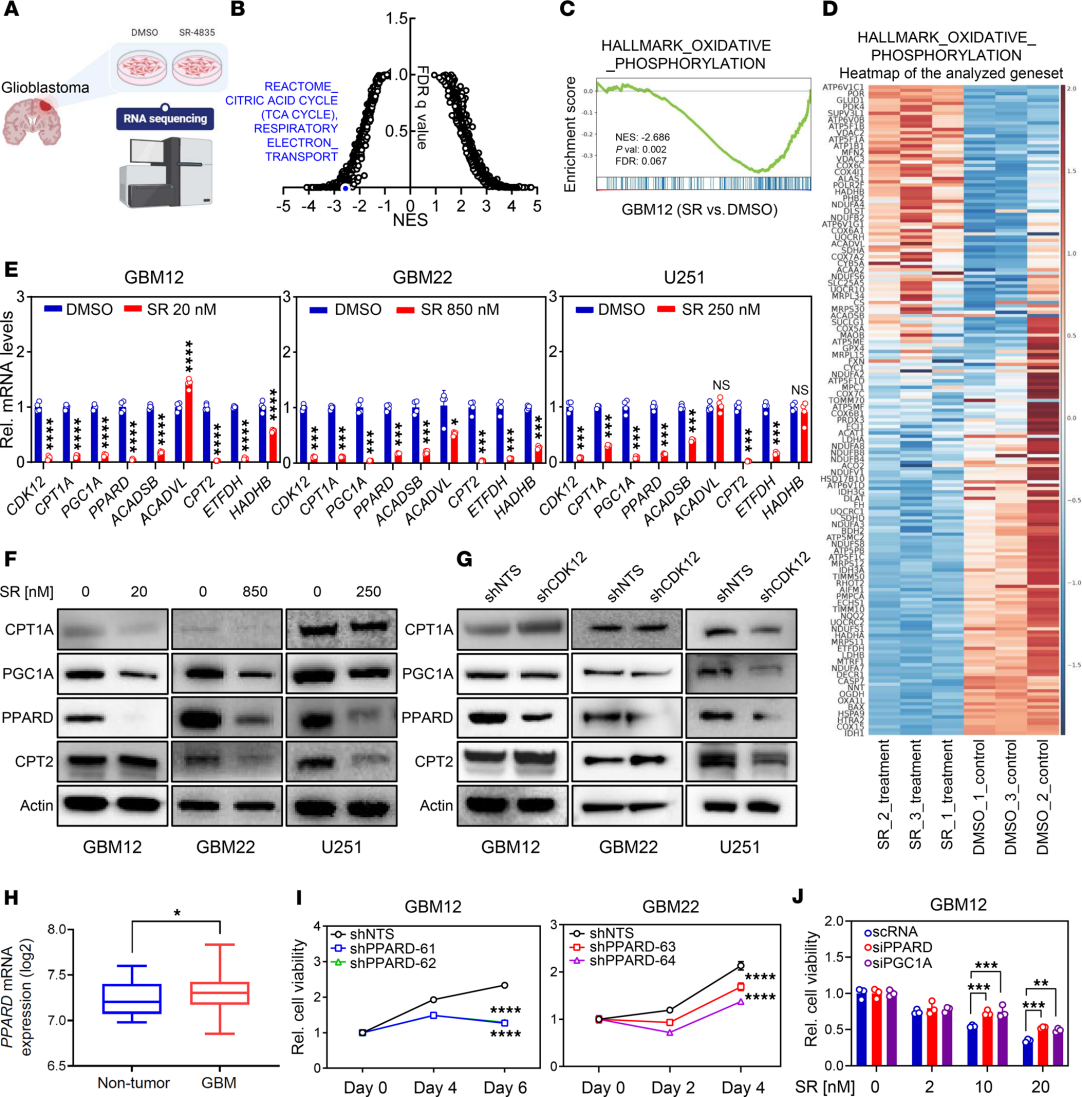
CDK12 inhibition impacts oxidative phosphorylation, influencing gene expression related to the TCA cycle, respiratory complexes, and fatty acid oxidation in GBM cells. (**A**) Schematic experimental design and RNA-seq sample information. (**B**) This plot shows the normalized enrichment score (NES) of Reactome_Citric acid cycle (TCA cycle) and Respiratory Electron_Transport gene sets derived from GSEA. (**C** and **D**) GSEA and heatmap of Oxidative Phosphorylation genes in RNA-seq data for DMSO and SR treatment. (**E** and **F**) The qPCR and Western blot results of oxidative phosphorylation– and fatty acid oxidation–related genes after SR treatment in GBM12, GBM22, and U251 cells. The qPCR data are presented as mean ± SD. (**G**) Western blot results of oxidative phosphorylation– and fatty acid oxidation–related genes after loss of CDK12 in GBM12, GBM22, and U251 cells. (**H**) PPARD mRNA expression (log_2_) in non-tumor and GBM tissues. Data are presented as box-and-whisker plots; the horizontal line within each box represents the median value, and the whiskers denote the minimum and maximum values. Statistical significance was determined using a Student’s *t* test (*P < 0.05). (**I**) Viability of GBM12 cells expressing shPPARD (61–64) compared to shNTS was assessed for 0, 2, 4, or 6 days. Data are presented as mean ± SD. (**J**) GBM12 cells were transfected with scrambled siRNA (scRNA) or siRNAs against PPARD and PGC1A. Thereafter, cells were exposed to increasing concentrations of SR-4835 and analyzed for cellular viability. Data are presented as mean ± SD. *P < 0.05; **P < 0.01; ***P < 0.001; ****P < 0.0001 by unpaired, 2-tailed *t* test (**E** and **H**), 1-way ANOVA with Dunnett’s multiple-comparison test (**I**), or 2-way ANOVA with Tukey’s multiple-comparison test (**J**).

To investigate how CDK12 inhibition induces GBM cell death, we performed RNA-seq on GBM12 cells treated with SR-4835 or vehicle (Figure 2, A–D). CDK12 inhibition profoundly downregulated genes involved in oxidative phosphorylation, including TCA cycle enzymes (e.g., *IDH3*, *OGDH*, *PDHA*) and components of the electron transport chain. Fatty acid oxidation–related transcripts were also suppressed (Figure 2, B–D). qPCR confirmed significant downregulation of key metabolic regulators *PGC1A* and *PPARD* across GBM lines, with GBM12 cells showing strong suppression at just 20 nM SR-4835 (Figure 2E). Western blots validated reduced PGC1A and PPARD protein levels in GBM12, GBM22, and U251 cells treated with SR-4835; shCDK12 knockdown also reduced PPARD in all 3 lines and PGC1A in U251 (Figure 2, F and G). PPARD mRNA expression was significantly elevated in GBM compared with non-tumor tissues in the Rembrandt cohort (Gene Expression Omnibus [GEO] accession GSE108476) (Figure 2H). Silencing *PPARD* or *PGC1A* in GBM12 and GBM22 reduced proliferation, with a more pronounced effect for *PGC1A* in GBM12 (Figure 2I and Supplemental Figure 2, A–C). Importantly, knockdown of *PPARD* or *PGC1A* attenuated the growth-inhibitory effect of SR-4835, indicating their role in mediating CDK12-dependent metabolic vulnerability (Figure 2J). Together, these data highlight CDK12 as a central regulator of oxidative metabolism in GBM.

### CDK12 regulates intrinsic apoptosis modulators.

**Figure 3:**
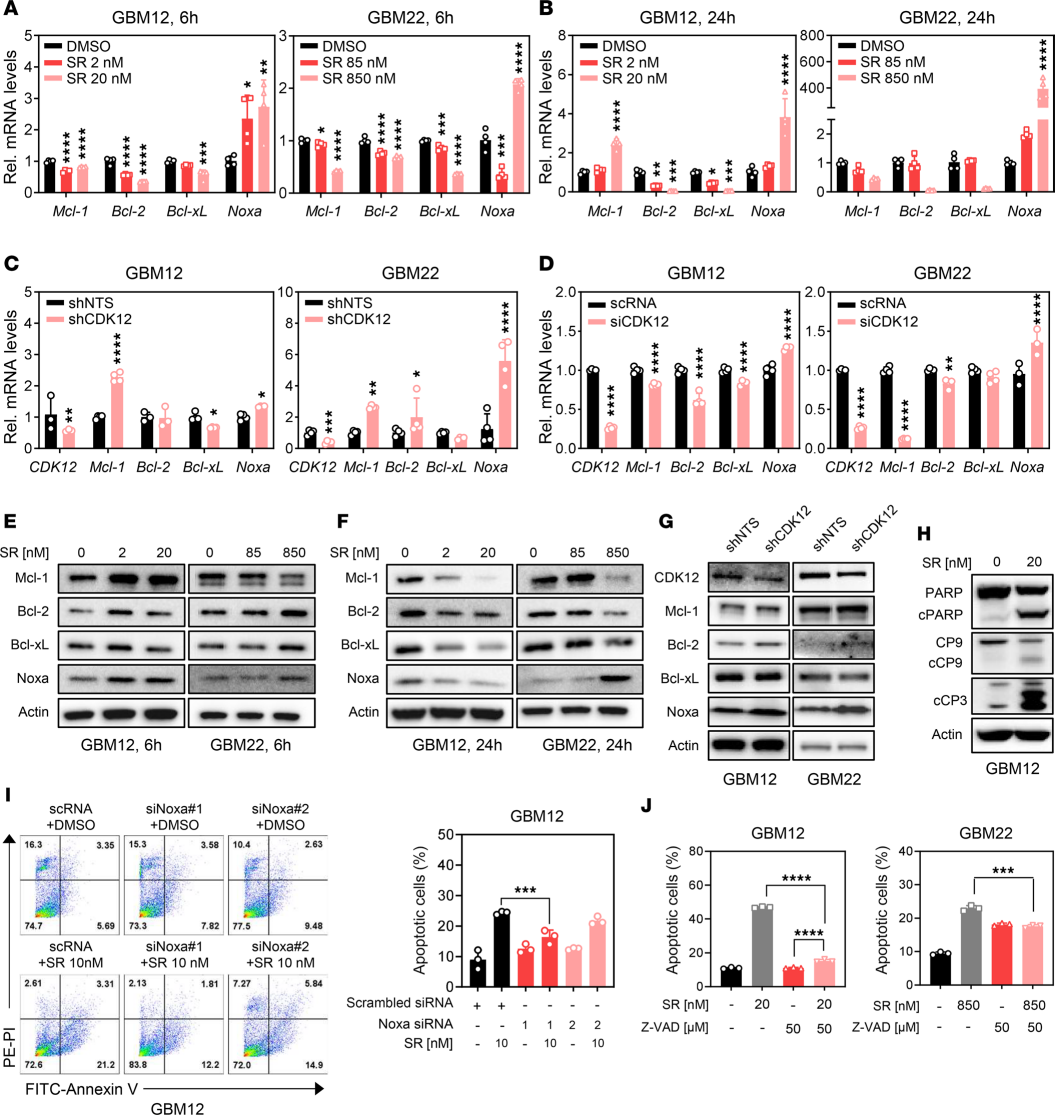
Inhibition of CDK12 influences apoptosis pathways, through downregulation of antiapoptotic Bcl-2 family and upregulation of proapoptotic Noxa, thereby enhancing caspase activation and apoptosis in GBM cells. (**A** and **B**) The qPCR analysis of apoptosis-related genes of GBM12 and GBM22 cells treated with increasing concentrations of SR for 6 hours and 24 hours. qPCR data are presented as mean ± SD. (**C** and **D**) The qPCR analysis in apoptosis-related genes of loss of CDK12, and siCDK12 in GBM12 and GBM22 cells. The qPCR data are presented as mean ± SD. (**E**–**G**) GBM12 and GBM22 cells were treated with SR for 6 or 24 hours, lysed, and subjected to Western blot analysis using the indicated antibodies to assess the expression of antiapoptotic Bcl-2 family proteins (Mcl-1, Bcl-xL, and Bcl-2) and the proapoptotic protein (Noxa). Representative immunoblots are shown. (**H**) Western blot results after SR treatment using anti-PARP, anti–cleaved caspase-9, and anti–cleaved caspase-3. (**I**) GBM12 cells were transfected with siNoxa_1 and 2 and treated with SR for 72 hours and analyzed by flow cytometry using annexin V/PI staining. The bar graph shows the percentage of apoptotic cells. Data are presented as mean ± SD. (**J**) GBM12 and GBM22 cells were treated with SR-4835, Z-VAD (50 μM), or a combination of both. Apoptosis was assessed by flow cytometry using annexin V/PI staining. The percentage of apoptotic cells was quantified. Data are presented as mean ± SD. *P < 0.05; **P < 0.01; ***P < 0.001; ****P < 0.0001 by 1-way ANOVA with Dunnett’s multiple-comparison test (**A**, **B**, **I**, and **J**) or unpaired, 2-tailed *t* test (**C** and **D**).

Beyond metabolic disruption, CDK12 inhibition by SR-4835 also impacted apoptosis and the integrated stress response. qPCR and Western blot analyses revealed reduced mRNA levels of antiapoptotic Bcl-2, Bcl-xL, and Mcl-1, alongside increased Noxa expression (Figure 3, A and B, and Supplemental Figure 3A). Similar changes were observed in shCDK12 and siCDK12 GBM cells (Figure 3, C and D). Western blot confirmed consistent Noxa protein upregulation following SR-4835 treatment and CDK12 knockdown (Figure 3, E–G, and Supplemental Figure 3B), indicating a shift toward a proapoptotic state. SR-4835 also activated initiator and effector caspases and induced PARP cleavage, hallmark features of apoptosis (Figure 3H). While Noxa levels increased up to 24 hours in most GBM lines, they later declined — likely due to its interaction with and degradation alongside Mcl-1, as reported previously (43). These findings suggest that CDK12 inhibition induces intrinsic apoptosis through modulation of Bcl-2 family members.

### Noxa mediates CDK12 inhibition–induced, caspase-dependent apoptosis.

Given the upregulation of proapoptotic Noxa, we investigated its role in CDK12 inhibition–induced cell death. Silencing *Noxa* via siRNA in GBM12 and U251 cells significantly reduced SR-4835–induced apoptosis, indicating Noxa is a key mediator of this response (Figure 3I and Supplemental Figure 3, C–F). CDK12 inhibition–induced apoptosis was also caspase dependent. In GBM12 and GBM22 cells, the pan-caspase inhibitor Z-VAD significantly reduced apoptosis triggered by SR-4835 (20 nM in GBM12; 850 nM in GBM22), as shown by decreased annexin V/PI staining (Figure 3J). Similarly, Z-VAD rescued apoptosis induced by shCDK12 in GBM22 (Supplemental Figure 3G). These findings show that both pharmacologic and genetic CDK12 inhibition induce Noxa-driven, caspase-dependent apoptosis in GBM cells.

### CDK12 regulates cellular respiration and affects glucose utilization in PDX GBM cells.

**Figure 4:**
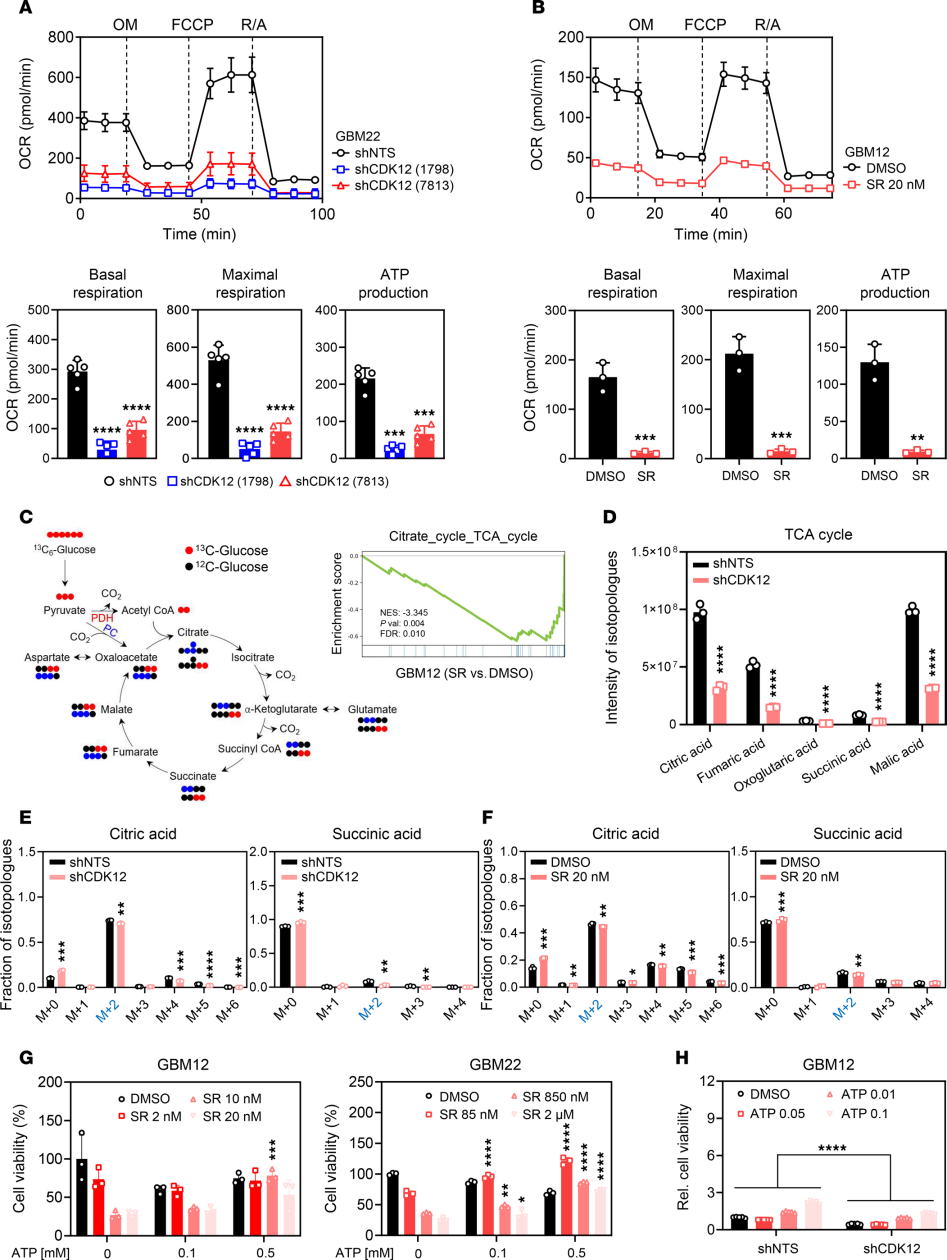
CDK12 regulates cellular respiration and glucose metabolism in GBM cells. (**A**) Seahorse mitochondrial stress assay was conducted on GBM22 cells expressing shNTS or shCDK12 (nos. 1798 and 7813). OM, oligomycin; FCCP, carbonyl cyanide-4 phenylhydrazone; R/A, rotenone/antimycin. Data are presented as mean ± SD. ***P < 0.001; ****P < 0.0001 by 1-way ANOVA with Dunnett’s multiple-comparison test. (**B**) Seahorse mitochondrial stress assay of GBM12 cells treated with DMSO or 20 nM SR for 24 hours. Data are presented as mean ± SD. **P < 0.01; ***P < 0.001 by unpaired, 2-tailed *t* test. (**C**) Left: A schematic representation of the main reactions involved in a tracer experiment. Right: GSEA analysis of Citrate_cycle_TCA_cycle in RNA-seq data for DMSO and SR treatment. (**D**) Relative intensities of TCA cycle metabolites (citric acid, fumaric acid, oxoglutaric acid, succinic acid, and malic acid) in shNTS and shCDK12 GBM cells. Cells were cultured in DMEM containing 25 mM U-^13^C_6_-glucose, 4 mM glutamine, and 1.5% dialyzed FBS (*n* = 3, independent samples). Data are presented as mean ± SD. **P < 0.01; ***P < 0.001; ****P < 0.0001 by unpaired, 2-tailed *t* test. (**E** and **F**) shNTS and shCDK12 cells were cultured in DMEM containing 25 mM U-^13^C_6_-glucose, 4 mM glutamine, and 1.5% dialyzed FBS for 24 hours (*n* = 3, each group). GBM12 cells were treated with 20 nM SR in DMEM containing 25 mM U-^13^C_6_-glucose, 4 mM glutamine, and 1.5% dialyzed FBS for 24 hours. Shown are fractions of the isotopologues for each metabolite (*n* = 3, each group). *P < 0.05; **P < 0.01; ***P < 0.001 by unpaired, 2-tailed *t* test. (**G**) GBM12 and GBM22 cells were treated with SR, followed by various concentrations of ATP to assess cell viability. Data are presented as mean ± SD. *P < 0.05; **P < 0.01; ***P < 0.001; ****P < 0.0001 by unpaired, 2-tailed *t* test. (**H**) GBM12 shNTS and shCDK12 (no. 1798) cells were treated with ATP at various concentrations for 48 hours, and cell viability was evaluated. Data are presented as mean ± SD. ****P < 0.0001 by 2-way ANOVA followed by Tukey’s multiple-comparison test.

Transcriptomic analysis of GBM cells lacking CDK12 revealed a significant downregulation of genes involved in the electron transport chain, suggesting impairment in oxidative phosphorylation and mitochondrial respiration. To further explore this, we utilized extracellular flux analysis with the Seahorse mitochondrial stress test to quantify key parameters of mitochondrial function, including basal respiration, ATP production, and maximal respiratory capacity, in GBM cells with and without CDK12 deficiency. Our Seahorse metabolic analyses in GBM12, GBM22, and U251 cells revealed that genetic knockdown of CDK12 using either of 2 distinct shRNAs resulted in impaired mitochondrial function. Specifically, we observed decreases in basal respiration, maximal respiratory capacity, and ATP production coupled to oxidative phosphorylation in the CDK12-deficient cells (Figure 4A and Supplemental Figure 4, A and B). These effects were most pronounced in the GBM22 line. Next, we sought to determine whether pharmacological inhibition of CDK12 would recapitulate the mitochondrial dysfunction observed in our genetic loss-of-function experiments. To this end, we exposed GBM12, GBM22, and U251 cells to a low nanomolar dosage of SR-4835. Consistent with our previous findings, we observed a substantial suppression of mitochondrial respiration upon treatment with SR-4835, including decreased basal respiration, maximal respiratory capacity, and ATP production coupled to oxidative phosphorylation (Figure 4B and Supplemental Figure 4, C and D). In addition, genetic knockdown of CDK13 also led to a reduction in oxidative phosphorylation, along with decreases in basal respiration, maximal respiratory capacity, and ATP production (Supplemental Figure 4E). These results suggest that pharmacological targeting of CDK12 is sufficient to impair mitochondrial function in GBM cells, further underscoring the critical role of CDK12 in regulating cellular energetics.

Given the strong impact of SR-4835 on cellular respiration, we performed metabolite screening and tracing with a focus on the TCA cycle and related pathways (Figure 4C). Acetyl-CoA enters the cycle to form citrate, which is sequentially converted into 2-oxoglutarate, succinate, fumarate, malate, and oxaloacetate, thus completing the cycle. The TCA cycle generates NADH_2_ and FADH_2_, which fuel the electron transport chain, with oxygen as the final electron acceptor and ATP synthesized via complex V (Figure 4C). Beyond energy production, the TCA cycle intermediates support biosynthesis of amino acids, lipids, and nucleotides — essential for aggressive GBM growth. Targeting mitochondrial metabolism has shown promise in selectively killing GBM cells. A key adaptation in GBM is glutamine oxidation via glutamate dehydrogenase, which converts glutamate to 2-oxoglutarate, feeding the TCA cycle. Similarly, oxaloacetate replenishment via pyruvate carboxylase supports anaplerosis (Figure 4C). As anticipated, CDK12-deficient cells substantially reduced levels of key TCA metabolites, including fumarate, 2-oxoglutarate, succinate, and malate (Figure 4D). These reductions in TCA intermediates suggest that the compound’s pharmacological interference with CDK12 significantly impairs the key functions of the TCA cycle, such as energy generation and biosynthesis of macromolecules. These findings provided a solid foundation to further investigate the metabolic effects of this compound through carbon tracing analyses, utilizing uniformly labeled U-^13^C-glucose as a tracer (Figure 4, E and F). This approach enabled a more comprehensive understanding of how SR-4835 disrupted the TCA cycle and the broader metabolic adaptations of GBM cells, offering valuable insights into the metabolic vulnerabilities of these aggressive brain tumor cells. The glucose carbon tracing studies revealed a substantial decrease in the amount of ^13^C incorporation from glucose into key TCA cycle intermediates, such as citrate and succinate, following treatment with SR-4835 (Figure 4, E and F, and Supplemental Figure 4F). A decrease in the labeling of nucleotides, particularly energy-rich ones like ATP (Supplemental Figure 4F), was observed. This reduction was consistent with our findings, which revealed a decrease in the labeling of substrates essential for both purine and pyrimidine synthesis. This decrease began at the fundamental nucleotide backbone, specifically ribose-5-phosphate, the final product of the pentose phosphate pathway. Consistently, we noted a reduction in labeling of aspartate and as well as serine and glycine (Supplemental Figure 4F). These effects on metabolism are likely to contribute to a suppressed synthesis of both nucleotide classes, affecting DNA synthesis as well as repair.

The glutamine tracing experiments revealed that the CDK12 inhibitor SR-4835 impacted glutamine oxidation and metabolism, indicating a crucial role for CDK12 in regulating both glucose and glutamine metabolic pathways (Supplemental Figure 4G). Notably, a slight decrease in the m+5 glutamic acid and oxoglutarate isotopologues was observed (Supplemental Figure 4G). One of the most significant changes identified was an enhancement in glutamine-driven citrate labeling, primarily manifested by an increase in the m+5 citrate isotopologue (Supplemental Figure 4G). This observation suggests a reverse flow of glutamine carbons, a phenomenon previously described as reductive carboxylation, which may occur in the context of mitochondrial stress. Collectively, these findings suggest that the pharmacological inhibition of CDK12 by SR-4835 significantly impaired the metabolic flux through the TCA cycle, thereby disrupting energy generation and biosynthetic processes in the GBM cells.

To validate the findings obtained with the CDK12 inhibitor SR-4835, we performed genetic loss-of-function experiments using shRNA specifically targeting CDK12. These experiments phenocopied the effects observed with pharmacological inhibition. Stable isotope tracing with ^13^C-glucose revealed a marked reduction in carbon incorporation into key TCA cycle intermediates, including citrate and succinate (Figure 4D and Supplemental Figure 4H). Additionally, labeling of amino acids derived from TCA cycle intermediates was similarly decreased (Supplemental Figure 4H). CDK12 knockdown in GBM22 cells also impaired glucose carbon flux through glycolysis, as evidenced by reduced labeling of both pyruvate and lactate (Supplemental Figure 4H). Furthermore, the 24-hour time interval was selected to capture enduring metabolic changes rather than transient fluctuations. We acknowledge that shorter timeframes could yield supplementary insights, and we recognized this as a limitation.

Next, we investigated whether the effects of CDK12 loss of function on metabolism are driver events or merely passenger effects. To narrow this down, we performed rescue experiments involving ATP. We found that increasing ATP levels was able to rescue the loss of viability mediated by CDK12 loss of function in GBM12, GBM22, and U251 GBM cell lines (Figure 4, G and H, and Supplemental Figure 4, I and J). This suggests that the reduced oxidative energy metabolism caused by CDK12 inhibition is a key driver for the loss of viability observed in these GBM cells, rather than merely a passenger effect. These results highlight the critical role of CDK12 in maintaining the metabolic homeostasis required for the survival and proliferation of GBM cells.

### GSK3β directly interacts with CDK12 and modulates GBM metabolism and growth.

**Figure 5:**
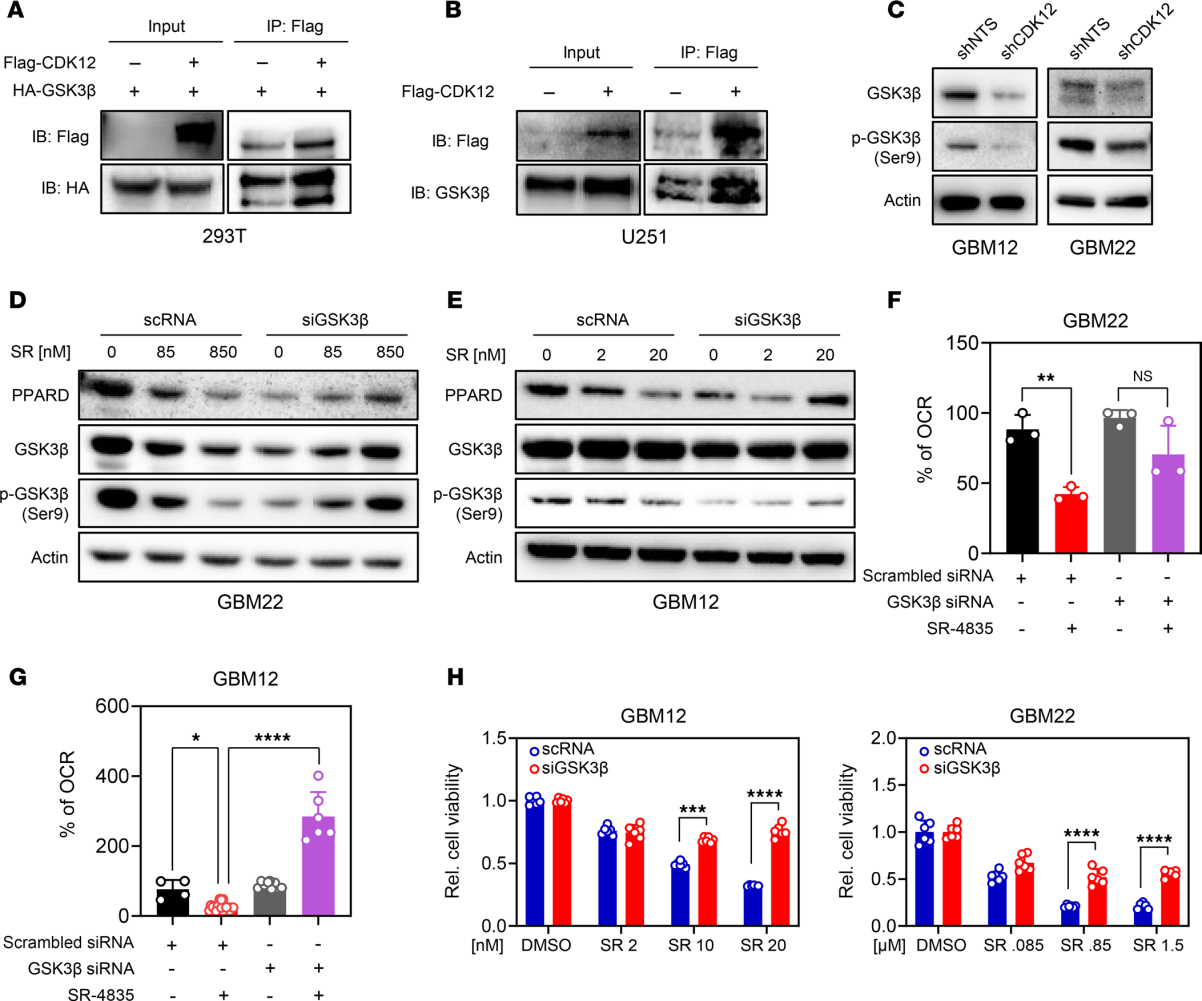
CDK12 regulates metabolism by interacting with GSK3β. (**A**) HEK293T cells were transfected with plasmids expressing HA-tagged GSK3β or a combination of HA-tagged GSK3B and FLAG-tagged CDK12 cDNA. Twenty-four hours after transfection, lysates were prepared and subjected to immunoprecipitation using anti-FLAG antibody. Input lysates and immunoprecipitates were analyzed using Western blotting with either anti-HA or anti-FLAG antibodies. Representative Western blots are presented. (**B**) U251 cells were transfected with plasmid expressing FLAG-tagged CDK12 cDNA. Twenty-four hours after transfection, lysates were prepared and subjected to immunoprecipitation using anti-FLAG antibody. Input lysates and immunoprecipitates were analyzed using Western blotting with either anti-FLAG or anti-GSK3β antibodies. Representative Western blots are presented. (**C**) GBM12 and GBM22 cells were transduced with either non-targeting or CDK12-specific shRNAs. Subsequently, cell lysates were harvested and analyzed by Western blotting for the expression of phosphorylated (Ser9) and total GSK3β. Actin was utilized as a loading control. (**D** and **E**) GBM22 and GBM12 cells were transfected with either non-targeting or GSK3β-specific siRNA. Subsequently, the cells were treated with increasing concentrations of SR-4835 and harvested for Western blot analysis to determine the expression of PPARD and total and p-GSK3β. Actin was used as a loading control. (**F** and **G**) GBM22 and GBM12 cells were transfected with either non-targeting or GSK3β-specific siRNA. Subsequently, the cells were treated with SR-4835 and subjected to extracellular flux analysis on the Seahorse analyzer to measure the oxygen consumption rate (OCR). The relative percentages of OCR are presented. Data are presented as mean ± SD. *P < 0.05; **P < 0.01; ****P < 0.0001 by 1-way ANOVA with Dunnett’s multiple-comparison test. NS, not significant. (**H**) GBM12 and GBM22 cells were transfected with non-targeting or GSK3β-specific siRNA and subsequently treated with increasing concentrations of SR-4835. Thereafter, cellular viability was assessed. Data are presented as mean ± SD. ***P < 0.001; ****P < 0.0001 by 2-way ANOVA with Tukey’s multiple-comparison test.

GSK3β, a kinase with tumor-suppressive roles, is often inactivated in cancer. Computational modeling predicted a direct interaction between GSK3β and CDK12, which we confirmed by coimmunoprecipitation; HA-GSK3β coprecipitated with FLAG-CDK12 only in cotransfected cells (Figure 5A). This interaction was also validated in U251 GBM cells, where FLAG-CDK12 pulled down endogenous GSK3β (Figure 5B). To assess functional relevance, we measured GSK3β activity via serine 9 phosphorylation. CDK12 knockdown in GBM12 and GBM22 cells reduced p-GSK3β (S9), indicating increased GSK3β activity (Figure 5C). Similarly, SR-4835 treatment decreased p-GSK3β in control siRNA cells, with a corresponding drop in PPARD levels. However, silencing GSK3β blocked SR-4835’s ability to reduce PPARD, suggesting GSK3β mediates this effect (Figure 5, D and E). Silencing GSK3β also rescued the SR-4835–induced reduction in oxygen consumption in both GBM12 and GBM22 cells (Figure 5, F and G). Furthermore, GSK3β knockdown diminished the antiviability effects of SR-4835, confirming its role in CDK12-regulated cell metabolism and survival (Figure 5H). These findings establish GSK3β as a functional CDK12 interactor and key mediator of its metabolic and growth-regulatory effects in GBM.

### CDK12 inhibition enhances the efficacy of standard-of-care therapies in GBM cells.

**Figure 6:**
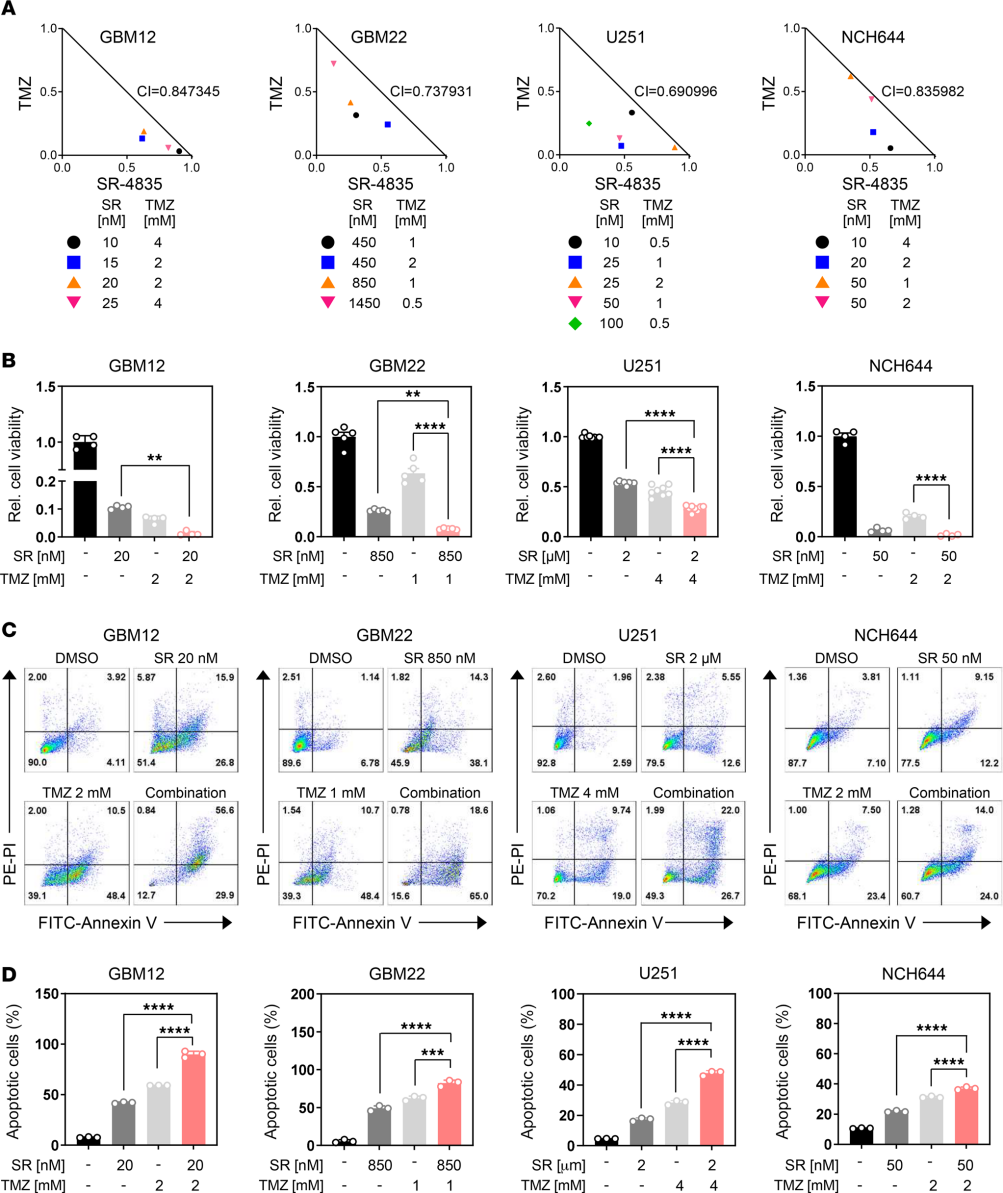
The combination treatment of SR-4835 and TMZ induces a synergistic growth reduction in GBM cells. (**A** and **B**) GBM12, GBM22, U251, and NCH644 cells were treated with SR and TMZ for 72 hours. Cellular viability was conducted, and synergism analysis was performed. Isobolograms are shown. (**C** and **D**) GBM12, GBM22, U251, and NCH644 cells were treated with SR, TMZ, and a combination for 72 hours and were analyzed by flow cytometry with annexin V/PI staining. The bar graph shows the percentage of apoptotic cells. Data are presented as mean ± SD. **P < 0.01; ***P < 0.001; ****P < 0.0001 by 1-way ANOVA with Dunnett’s multiple-comparison test.

Standard GBM treatment includes surgical resection, radiation, and the DNA-alkylating agent TMZ. However, TMZ efficacy is often limited by GBM’s metabolic adaptability. Given CDK12’s role in maintaining metabolic homeostasis, we hypothesized that its inhibition could enhance TMZ’s antitumor effects. Using GBM12, GBM22, NCH644, and U251 cell lines, we tested combinations of SR-4835 (CDK12 inhibitor) and TMZ across clinically relevant doses. Chou-Talalay analysis revealed synergistic effects in all lines, with combination index (CI) values less than 1, indicating enhanced efficacy (Figure 6, A and B). Flow cytometry analysis using annexin V/PI staining showed that the combination treatment significantly increased apoptosis and overall cell death across the GBM cell panel compared with either agent alone (Figure 6, C and D). These findings suggest that CDK12 inhibition sensitizes GBM cells to TMZ, providing a strong rationale for combining CDK12 inhibitors with standard therapy to overcome treatment resistance.

### CDK12 inhibition impairs growth of orthotopic PDX GBM tumors in vivo.

**Figure 7:**
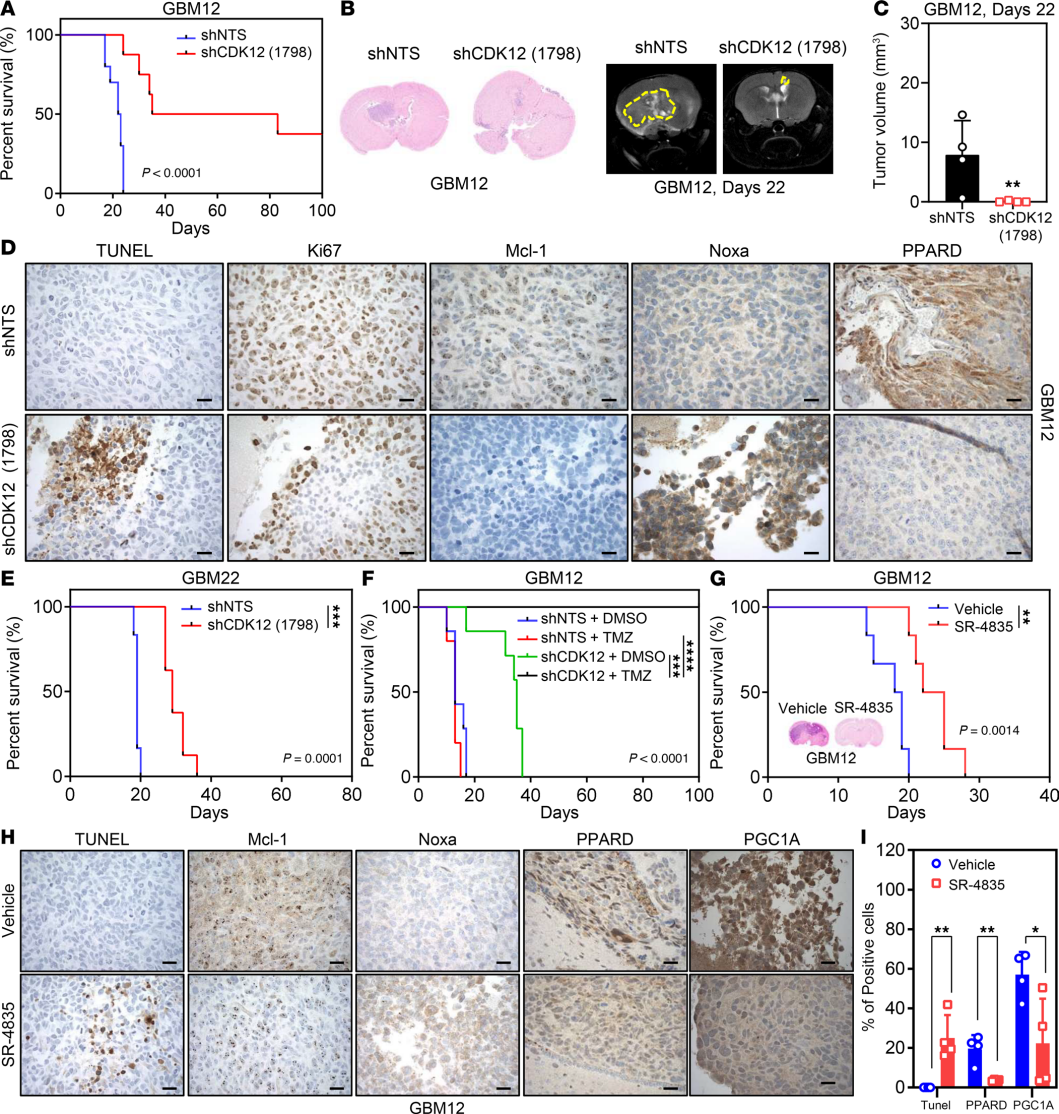
Combination treatment with CDK12 inhibition and TMZ leads to regression of intracranial GBM PDX tumors. (**A**) The Kaplan-Meier survival curves were employed to determine overall survival between GBM12 shNTS and shCDK12 (no. 1798) mice. The median survival in the shNTS (*n* = 10) and shCDK12 (*n* = 8) groups was 22.5 and 59 days, respectively. A log-rank test was utilized to assess the statistical significance of the observed differences (*P* < 0.0001). (**B**) Representative H&E staining images and MRI of GBM12 shNTS and shCDK12 mouse brains at 22 days (Bruker BioSpec, 9.4 Tesla). (**C**) The bar graph shows mouse tumor volumes of brains in **B**, which were analyzed by Analyze 14.0 (www.analyzedirect.com). Data are presented as mean ± SD. **P < 0.01 by unpaired, 2-tailed *t* test. (**D**) Tumors were fixed and stained with TUNEL and for Ki67, Mcl-1, Noxa, and PPARD. Scale bar: 30 μm. (**E**) Kaplan-Meier survival curves were employed to determine overall survival between GBM22 shNTS and shCDK12 (no. 1798) groups. The median survival in shNTS (*n* = 6) and shCDK12 (no. 1798) (*n* = 8) groups was 19 and 29 days, respectively (log-rank, *P* = 0.0001). (**F**) The median survival in the shNTS+DMSO (*n* = 7, blue line), shNTS+TMZ (*n* = 5, red line), shCDK12+DMSO (*n* = 7, green line), and shCDK12+TMZ (*n* = 7, black line) groups were 13, 13, 35, and not-reached days, respectively (log-rank, *P* < 0.0001). (**G**) GBM12 PDX cells were intracranially injected into the brains of mice and then treated with vehicle (DMSO) or SR-4835 by intraperitoneal injection for 2 weeks. The median survival in DMSO (*n* = 6) and SR (*n* = 6) groups was 18.5 and 23.5 days, respectively (log-rank, *P* < 0.0014). Representative H&E staining images of GBM12 DMSO and SR group mouse brains. (**H**) Tumors from the experiment in **G** were fixed and stained with TUNEL and for Mcl-1, Noxa, PPARD, and PGC1A. Scale bar: 30 μm. (**I**) Quantification of IHC staining intensity for TUNEL, PPARD, and PGC1A presented as a bar graph. Data are presented as mean ± SD. *P < 0.05; **P < 0.01 by unpaired, 2-tailed *t* test.

To evaluate the therapeutic potential of CDK12 inhibition in vivo, we used intracranial PDX models with GBM12 and GBM22 cells in immunocompromised mice. GBM12 xenografts with CDK12 knockdown showed significantly reduced tumor growth by MRI and prolonged survival compared with controls (Figure 7, A–C). Histological analysis revealed increased TUNEL positivity and decreased Ki67, indicating enhanced apoptosis and reduced proliferation (Figure 7D). CDK12 knockdown also increased proapoptotic Noxa, decreased antiapoptotic Mcl-1, and reduced PPARD expression (Figure 7D). Similarly, CDK12 knockdown in GBM22 xenografts extended survival (Figure 7E). We next tested pharmacologic inhibition using SR-4835 in GBM12-bearing mice. Treatment significantly reduced tumor growth and improved survival (Figure 7G). SR-4835 increased Noxa, decreased Mcl-1, and enhanced TUNEL staining, indicating apoptosis in vivo (Figure 7, H and I, and Supplemental Figure 5A). SR-4835 also reduced PPARD and PGC1A expression (Figure 7, H and I). These results demonstrate that both genetic and pharmacologic inhibition of CDK12 suppresses GBM tumor growth in orthotopic PDX models, supporting CDK12 as a promising therapeutic target in GBM.

### CDK12 inhibition enhances TMZ efficacy in orthotopic GBM PDX models.

Building on our in vitro findings of synergy between SR-4835 and TMZ, we tested whether CDK12 inhibition could overcome TMZ resistance in vivo using the aggressive GBM12 orthotopic PDX model. GBM12 cells expressing non-targeting or CDK12-targeting shRNA were implanted intracranially in nude mice. After tumor establishment, mice were assigned to 4 groups: (a) non-targeting plus vehicle, (b) non-targeting plus TMZ, (c) CDK12 shRNA plus vehicle, and (d) CDK12 shRNA plus TMZ. Survival was monitored (Figure 7F). Mice bearing non-targeting tumors died rapidly, with or without TMZ. In contrast, CDK12 knockdown significantly extended survival, even without TMZ. Most notably, the combination of CDK12 knockdown and TMZ led to complete tumor regression, with long-term survival in all mice (Figure 7F). These results demonstrate that CDK12 inhibition can overcome TMZ resistance and dramatically enhance therapeutic efficacy in a clinically relevant GBM model.

## Discussion

GBM remains a major therapeutic challenge with an urgent need for improved outcomes. Our study reveals that CDK12 is upregulated in GBM and correlates with poor patient survival. The selective CDK12 inhibitor SR-4835 effectively killed GBM cells, including resistant mesenchymal subtypes, at low concentrations, while sparing normal astrocytes, demonstrating tumor-specific toxicity (38–42). SR-4835 shows superior in vivo potential compared with earlier CDK inhibitors like THZ531 (42). Similar progress has been made with CDK7 inhibitors. THZ1, which targets CDK7, also inhibits CDK12/13, but has limited in vivo use (34–37). This led to the development of selective inhibitors like YKL-5-124, effective alone or in combination against solid tumors (36). Our data highlight CDK12 as a promising therapeutic target in GBM. Strikingly, CDK12 inhibition combined with TMZ eradicated tumors in all treated mice, a rare outcome in preclinical GBM models. CDK12 has been minimally studied in GBM, with only one prior report linking it to vasculogenic mimicry using subcutaneous, non-patient-derived models (44). Only a few studies have achieved similar in vivo efficacy, such as those using GL261 models responsive to anti–PD-1/PD-L1 and anti-CTLA4 therapies (45–47). However, GL261 poorly reflects human GBM due to its mutational profile and artificial carcinogen induction (48). Additionally, the use of fluorescent markers (e.g., GFP) in syngeneic models may trigger immune responses, potentially overstating therapeutic efficacy (49, 50). These limitations highlight the importance of patient-derived models. It remains an open question whether CDK12 inhibition could enhance antitumor immunity in GBM. Notably, CDK7 inhibition has been shown to boost anti–PD-1 responses in lung cancer models (36), suggesting a possible parallel worth exploring in GBM.

While the Warburg effect facilitates rapid glucose processing and NAD+ regeneration, oxidative metabolism remains essential for GBM and other cancers by supplying TCA cycle intermediates for biosynthesis. In oxygen-rich tumor regions, oxidative metabolism may be especially advantageous due to its higher energy yield compared with glycolysis. Unexpectedly, our RNA-seq and metabolite tracing data revealed that CDK12 significantly regulates oxidative metabolism in GBM models, beyond its known roles in transcription and cell cycle control. CDK12 reduces glucose-derived carbon entry into the TCA cycle, lowering biosynthesis linked to nucleotide production. Notably, active CDK12 also suppresses reductive carboxylation, a glutamine-dependent pathway critical for lipid synthesis and cancer proliferation (51, 52). These findings suggest a potential synergy between CDK12 inhibitors and glutaminase inhibitors. Regarding other metabolic pathways, CDK12 overexpression in breast tumors promotes the serine-glycine synthesis pathway, rendering these neoplasms more susceptible to methotrexate (53). Based on our findings, it is plausible that various cancers exhibit distinct metabolic requirements induced by CDK12. The underlying reasons for these differences warrant further investigation in future studies. In addition, future studies will be conducted to precisely narrow down the involved downstream mechanisms as to how GSK3β regulates metabolism.

Furthermore, our findings provide evidence that ATP, a key end product of cellular respiration, mitigates loss of viability caused by CDK12 loss of function. These investigations substantiate a hypothesis suggesting that the detrimental effect of CDK12 depletion is mediated through disruption of the electron transport chain and subsequent synthesis of ATP (oxidative phosphorylation). We also identified several mediators of CDK12’s effects on metabolism. Notably, we discovered that CDK12 facilitated the expression of the transcription factor PPARD, a well-known driver of oxidative metabolism (25), which is typically mediated through enhanced β oxidation. To the best of our knowledge, this observation has not been made before, and based on the available literature, this connection appears somewhat unexpected.

We also uncovered a previously uncharacterized functional interaction between CDK12 and GSK3β. Our data suggest that CDK12 positively regulates the inhibitory phosphorylation of GSK3β, thereby restraining its kinase activity. Inhibition or genetic depletion of CDK12 leads to reduced Ser9 phosphorylation and subsequent activation of GSK3β, indicating a regulatory relationship between the 2 proteins. This activation of GSK3β has several downstream consequences in our model systems. First, we observed that CDK12 influences the expression of the metabolic transcription factor PPARD in a GSK3β-dependent manner. Second, CDK12 inhibition reduced oxidative phosphorylation, as measured by oxygen consumption rate (OCR), and this metabolic effect was at least partially reversed by siRNA-mediated knockdown of GSK3β, suggesting that CDK12 modulates mitochondrial respiration through GSK3β signaling. Third, we found that the impact of CDK12 on GBM cell proliferation also depends on GSK3β activity. Together, these findings support a model in which CDK12 regulates a GSK3β/PPARD signaling axis that influences both transcriptional control of metabolism and mitochondrial function. However, we acknowledge that dedicated gain- and loss-of-function experiments for GSK3β and PPARD under CDK12-deficient conditions were not performed in this study, and further work is required to fully elucidate the mechanistic hierarchy and causal relationships within this pathway.

Disruption of oxidative phosphorylation activates the integrated stress response. We found that CDK12 inhibition triggers this response by upregulating stress-related transcription factors ATF3 and ATF4 (54), along with the proapoptotic Bcl-2 family member Noxa; we believe this is a novel finding in the context of CDK12 signaling. Antiapoptotic proteins like Mcl-1 were downregulated at later time points, shifting the apoptotic balance toward cell death. This balance between pro- and antiapoptotic Bcl-2 proteins influences cancer cell survival and response to therapy. Our data show that intrinsic apoptosis is the primary mechanism of cell death following CDK12 loss, evidenced by annexin V positivity and caspase activation in GBM cells. Other forms of cell death may occur in specific contexts but appear secondary. These findings reveal a new mechanism by which CDK12 limits stress response and apoptosis by regulating oxidative phosphorylation. While little is known about CDK12’s metabolic role, related kinases like CDK8 and CDK6 have been implicated in regulating glycolysis and adipocyte metabolism, respectively (55).

CDK12 signaling may influence the immune landscape of GBM. Single-cell RNA-seq studies reveal that GBMs are surrounded by an immunosuppressive environment dominated by myeloid-derived suppressor cells (MDSCs), M2 macrophages, and regulatory T cells, all of which hinder T cell activity (56–61). Given MDSCs’ reliance on oxidative metabolism, CDK12 inhibition may impair their function and promote T cell–mediated GBM killing. In prostate cancer, CDK12 loss defines an immunogenic subtype characterized by neoantigen production and increased sensitivity to immune checkpoint inhibitors, including anti–PD-1 therapy (62). This raises the possibility that CDK12 inactivation could also enhance GBM immunogenicity, although this remains to be tested. Importantly, CDK12 inhibition with SR-4835 has been shown to enhance anti–PD-1 efficacy in colorectal and breast cancer models by increasing CD8^+^ T cell infiltration into tumors (63, 64). These findings support further investigation into CDK12 as a modulator of immune response and a potential target to improve immunotherapy in GBM.

In summary, our research has shown that by disrupting CDK12, we can significantly enhance the efficacy of TMZ to completely eradicate tumors in animal models. Our work emphasizes the important role of CDK12 in regulating oxidative energy metabolism and oncogenesis by affecting GSK3β and PPARD. It also highlights the potential for targeting CDK12 as a therapeutic strategy, not only for GBM, but across different types of cancer. These findings present new opportunities for developing interventions aimed at CDK12 in GBM, with the potential to greatly improve patient outcomes.

## Methods

### Sex as a biological variable.

We did not analyze sex as a biological factor in this study. This research predominantly centered on female mice and explored the impacts of vehicle, monotherapy, and combination pharmacological interventions.

### Cell cultures and growth conditions.

All the GBM cell lines were incubated at 37°C and were maintained in an atmosphere containing 5% CO_2_. GBM12, GBM22, and GBM43 cells were obtained from Jann Sarkaria (Mayo Clinic, Rochester, Minnesota, USA). U251 cells were purchased from Sigma-Aldrich (catalog CB_09063001). KNS42 cells were purchased from the Japanese Collection of Research Bioresources Cell Bank (JCRB, IFO50356). U87 cells were purchased from American Type Culture Collection (ATCC). And NCH644 stem cell–like glioma cells were purchased from Cell Line Services (catalog 820403). GBM PDX lines GBM12, GBM22, U251, KNS42, GBM39, GBM67, U87, and GBM43 cells were cultured in DMEM, 10% FBS, and 100 μg/mL Primocin (InvivoGen, ant-pm-1). NCH644 stem-like glioma cells were cultured in NeuroCult NS-A basal medium (human) (Stemcell Technologies, 05750) with NS-A proliferation supplement (Stemcell Technologies, 05753), 20 ng/mL EGF (Stemcell Technologies, 78006.1), 10 ng/mL bFGF (Stemcell Technologies, 78003.1), 0.2% Heparin (Stemcell Technologies, 07980), and 100 μg/mL Primocin. Astrocytes were purchased from ScienceCell Research Laboratories and cultured in DMEM, 10% FBS, 100 μg/mL Primocin, and N2 Supplement (Thermo Fisher Scientific, 17502048). For the treatment experiments, all of cells were cultured in DMEM containing 1.5% FBS and 100 μg/mL Primocin.

### Reagents.

SR-4835 (catalog S8894), TMX (catalog S1237), ATP disodium (catalog S1985), and Z-VAD-FMK (catalog 7023) were purchased from Selleckchem. The DMSO concentrations in the experimental treatments were maintained at levels below 0.1% (v/v).

### Plasmids and siRNAs.

The following plasmid constructs were purchased from Sigma-Aldrich: shCDK12-1798 (catalog TRCN0000001798), shCDK12-1799 (catalog TRCN0000001799), shCDK12-7811 (catalog TRCN0000237811), shCDK12-7813 (catalog TRCN0000237813), shPGC1A-65 (catalog TRCN0000001165), shPGC1A-66 (catalog TRCN0000001166), shPGC1A-86 (catalog TRCN0000364086), shPGC1A-87 (catalog TRCN0000364087), shPPARD-61 (catalog TRCN0000001661), shPPARD-62 (catalog TRCN0000001662), shPPARD-63 (catalog TRCN0000001663), and shPPARD-64 (catalog TRCN0000001664). The scramble shRNA (shNTS, non-target sequence) plasmid was purchased from Addgene (catalog 1864). The HA-GSK3β plasmid was purchase from Addgene, and the FLAG-CDK12 plasmid was purchased from VectorBuilder. Non-targeting siRNA-pool (scRNA) (catalog D-001810-10-20) and siCDK12 (catalog L-004031-00-0005) were purchased from Dharmacon. siPMAIP1-1 (siNoxa_1, catalog s10708) and siPMAIP1-2 (siNoxa_2, catalog s10709) were purchased from Thermo Fisher Scientific.

### Lentivirus transduction.

Lentivirus production was achieved through transfection of HEK293T cells with pDM2.G and psPAX2 plasmids, along with a lentivirus transfer plasmid for 72 hours. The viral particles were filtered through 0.45 μm surfactant-free cellulose acetate (SFCA) syringe filters (Thermo Fisher Scientific, 09-754-21), and concentrated with Amicon Ultra-15 (Merck, UFC910024) before they were used for transduction of target GBM cells. For generating target gene–knockdown cells, cells were infected in 8 μg/mL polybrene (Santa Cruz Biotechnology, sc-134220) and were selected with 0.5–2 μg/μL puromycin (Santa Cruz Biotechnology, sc-108071).

### Cell viability assay.

Cell viability experiments were performed on GBM22 and U251 GBM cell lines, seeding 3 × 10^3^ cells per well in a 96-well plate. Similarly, GBM12 cells were seeded at a density of 8 × 10^3^ cells per well in a 96-well format. Following a 24-hour or 72-hour incubation period in medium supplemented with 1.5% FBS, cell viability was assessed using the CellTiter-Glo Luminescent Cell Viability Assay (Promega, G7571), as per the manufacturer’s protocol. To evaluate cell viability in the ATP rescue experiments, we employed the CyQUANT Cell Proliferation Assay (Thermo Fisher Scientific, C7026).

### Flow cytometry.

Cells were seeded at a density of 4 × 10^4^ cells per well in a 12-well plate and incubated for 24 hours. Subsequently, the cells were stained with Annexin V–FITC/PI (BD Biosciences, 556420) according to the manufacturer’s instructions to determine the rate of apoptosis. Cells were analyzed using a BD Fortessa or BD FACSCanto II flow cytometer and the data were processed with FlowJo software (version 10.10.0; Tree Star).

### Western blotting and protein capillary electrophoresis.

Whole-cell protein samples were obtained using 1× Laemmli buffer containing β-mercaptoethanol and then subjected to thermal denaturation for 15 minutes at 95°C. Protein samples were loaded on 4%–12% gradient SDS-PAGE gels purchased from Invitrogen (catalog NP0321BOX) and transferred to PVDF membranes (Bio-Rad, 1620177). Imaging and quantification of the membranes were performed using the Azure C300 digital imaging system (Azure Biosystems), which provides high-resolution, sensitive detection and analysis of protein bands separated by gel electrophoresis. For protein capillary electrophoresis, samples were collected in protein lysis buffer. Samples were detected by using the Wes instrument (ProteinSimple) following manufacturer’s instructions. Antibodies against the following proteins were used: CDK12 (Cell Signaling Technology [CST], 11973), Mcl-1 (CST, 5453), Bcl-2 (clone D55G8, CST, 4223), Bcl-xL (clone 54H6, CST, 2764), Noxa (clone 114C307, Calbiochem, OP180), PARP (clone 46D11, CST, 9532), CCP9 (clone D2D4, CST, 7237), CCP3 (Asp175, CST, 9661), CPT2 (Invitrogen, PA5-12217), PGC1A (Novus Bio, NBP1-04676), PPARD (Abcam, ab23673), GSK3β (clone 27C10, CST, 9315), p-GSK3β (Ser9) (clone D85E12, CST, 5558), HA (CST, 3724S), FLAG (Sigma-Aldrich, F3165), β-actin (Sigma-Aldrich, A1978), anti–rabbit IgG (H+L), HRP (Thermo Fisher Scientific, 31460), and anti–mouse IgG (H+L), HRP (Thermo Fisher Scientific, 31430). For protein capillary electrophoresis, anti-CDK12 (CST, 11973) and Vinculin (Abcam, ab129002) were used. The secondary antibodies were anti-rabbit–HRP (ProteinSimple, 042-206) and anti-mouse–HRP (ProteinSimple, 042-205).

### qPCR analysis.

Total RNA was extracted using the miRNAeasy Mini Kit (QIAGEN, 217004), and 500 ng of the extracted RNA was reverse transcribed into cDNA by employing the cDNA synthesis kit from Origene (catalog NP100042). qPCR was conducted with Power SYBR Green PCR Master Mix (Applied Biosystems, 4367659), and the amplification protocol involved an initial denaturation step at 95°C for 10 minutes, followed by 40 cycles of 95°C for 15 seconds, 60°C for 30 seconds, and 72°C for 30 seconds, performed on a Quantabio qPCR instrument. The relative mRNA expression levels for the genes of interest were determined by normalization to 18S mRNA expression using the ΔΔCt method. The qPCR primer sequences are presented in Supplemental Table 1.

### RNA-seq.

Total RNA from each sample was extracted using the RNeasy isolation kit (Qiagen, 217004). RNA (100 ng) with RIN greater than 8 was mixed with the RNA library SIRV-Set1 (Iso Mix E0, E1, E2)-RNA seq according to the company’s instructions. The libraries were sequenced and analyzed with Element AVITI (Element Biosciences) at the Columbia University Genome Center. RNA-seq data analysis was performed by using Scidap (https://scidap.com/).

### Liquid chromatography, mass spectrometry, and isotope tracing.

Cells were seeded at 1 × 10^6^ cells per 10-cm cell culture dish and incubated for 24 hours. And then, for glucose tracing, cells were treated with DMEM without glucose, glutamine, or phenol red (Thermo Fisher Scientific, A1443001), containing 25 mM U-^13^C_6_ D-glucose (Cambridge Isotope Laboratories, Inc), 4 mmol/L glutamine (Thermo Fisher Scientific, 15410314), and 1.5% dialyzed FBS (Thermo Fisher Scientific, A3382001) for 24 hours. For glutamine tracing, cells were treated containing 25 mM D-glucose, 4 mmol/L U-^13^C_5_-glutamine (Cambridge Isotope Laboratories, Inc), and 1.5% dialyzed FBS (Thermo Fisher Scientific, A3382001) for 24 hours. Cells were analyzed for polar metabolites at Weill Cornell Metabolomics Core Facility.

### Extracellular flux analysis.

GBM cells were seeded at a density of 3 × 10^4^ per XFp, XFe24, and XFe96 cell culture microplates procured from Agilent. The cells were allowed to adhere for 24 hours, after which they were exposed to the target compounds for a subsequent 24-hour duration. The OCR was quantified using a Seahorse XFp, XFe24, XFe96 Analyzer (Agilent) and the Cell Mito-Stress Assay kit (Agilent, 103015–100) in the Seahorse XF base medium (Agilent, 102353–100), which contained 10 mM glucose, 2 mM glutamine, and 1 mM pyruvate. The metabolic compounds were injected sequentially as instructed by the manufacturer: 2 μM oligomycin (OM), 2 μM carbonyl cyanide-4 phenylhydrazone (FCCP), and 0.5 μM rotenone/antimycin (R/A).

### Orthotopic GBM PDX mouse model.

Either 1 × 10^5^ (experiment in Figure 7A) or 2 × 10^5^ (experiment in Figure 7F) GBM12 cells and 5 × 10^4^ GBM22 cells were stereotactically implanted into the brains of 4- to 6-week-old female nude mice (NCRNU-F sp/sp, CrTac:NCr-*Foxn1^nu^*), with the injection site located 3 mm lateral to bregma and 3 mm in depth. SR-4835 (5–7.5 mg/kg) and TMZ (50 mg/kg) were dissolved in a mixture containing DMSO (Sigma-Aldrich, D2438), Cremophor EL (Sigma-Aldrich, 61791–12–6), ethyl alcohol (Pharmco-Aaper, 200 proof), and PBS at the ratio 10:32:8:50 (v/v/v/v). The SR compound was administered via intraperitoneal injections 3 times per week for a duration of 2 weeks, while the TMZ treatment was given 5 times per week over the same 2-week period. The survival data was analyzed using Kaplan-Meier survival analysis techniques, and the statistical significance was assessed through the application of the log-rank test.

### TUNEL and immunohistochemistry.

The immunohistochemical assessment for TUNEL and Noxa was carried out in accordance with previously established protocols. Furthermore, the quantification of Mcl-1, Noxa (LSBio, LS-B184), and PPARD (Invitrogen, PA5-96377) involved the assignment of an immunohistochemical score that considered both the staining intensity (0: no staining, 1: weak staining intensity, 2: intermediate staining intensity, 3: strong staining intensity) and the percentage of positively stained cells (0: no cells labeled, 1: 1%–25%: cells labeled, 2: 25%–74%: cells labeled, 3: 75%–100% cells labeled).

### Statistics.

Statistical significance was evaluated by an unpaired, 2-tailed Student’s *t* test or 1-way ANOVA (for multiple comparisons) with Dunnett’s test or 2-way ANOVA (for multiple comparisons) with Tukey’s test using GraphPad Prism, version 10. Unless specified otherwise, 3 replicates were conducted. A significant level of *P* less than 0.05 was chosen. For analyzing drug synergism, CompuSyn software was used to calculate the CI (CI < 1; synergistic, CI = 1; additive and CI > 1; as antagonistic). IC_50_ values were determined through nonlinear regression.

### Study approval.

All procedures were done in accordance with Animal Welfare Regulations and approved by the Institutional Animal Care and Use Committee at the Columbia University Medical Center.

### Data availability.

The RNA-seq data generated in this study have been deposited in the NCBI GEO database under accession no. GSE306109. All data points shown in graphs and values behind reported means are provided in the Supporting Data Values.

## Author contributions

JYM and MDS conceived and designed the study. JYM, QG, ZZ, HOA, CS, and MDS developed the methodology. JYM, CS, QG, and MDS acquired data. JYM, CS, QG, ZZ, OAM, MAW, GKM, and MDS wrote, reviewed, and revised the manuscript. MDS supervised the study.

## Funding support

This work is the result of NIH funding, in whole or in part, and is subject to the NIH Public Access Policy. Through acceptance of this federal funding, the NIH has been given a right to make the work publicly available in PubMed Central.

- National Institute of Neurological Disorders and Stroke/NIH grants R01NS113793 and R21NS137660.
- NIH center grants P30CA013696 and S10RR027050 (to the Columbia University Medical Center Cancer Center Flow Core Facility).
- Columbia University W.P. Carey Residency Program (to ZZ).
- Columbia University Research Stabilization Fund grant.

## Supplementary Material

Supplemental data

Unedited blot and gel images

Supporting data values

## Version 1 09/25/2025

In-Press Preview

## Version 2 11/10/2025

Electronic publication

## References

[1] Ravi VM (2022). Spatially resolved multi-omics deciphers bidirectional tumor-host interdependence in glioblastoma. Cancer Cell.

[2] Chen Z (2020). Genetic driver mutations introduced in identical cell-of-origin in murine glioblastoma reveal distinct immune landscapes but similar response to checkpoint blockade. Glia.

[3] Kilian M (2022). MHC class II-restricted antigen presentation is required to prevent dysfunction of cytotoxic T cells by blood-borne myeloids in brain tumors. Cancer Cell.

[4] Gimple RC (2019). Glioma stem cell-specific superenhancer promotes polyunsaturated fatty-acid synthesis to support EGFR signaling. Cancer Discov.

[5] Mack SC (2018). Therapeutic targeting of ependymoma as informed by oncogenic enhancer profiling. Nature.

[6] Parsons DW (2008). An integrated genomic analysis of human glioblastoma multiforme. Science.

[7] Stupp R (2017). Effect of tumor-treating fields plus maintenance temozolomide vs maintenance temozolomide alone on survival in patients with glioblastoma: a randomized clinical trial. JAMA.

[8] Garofano L (2021). Pathway-based classification of glioblastoma uncovers a mitochondrial subtype with therapeutic vulnerabilities. Nat Cancer.

[9] Torrini C (2022). Lactate is an epigenetic metabolite that drives survival in model systems of glioblastoma. Mol Cell.

[10] Lin H (2017). Fatty acid oxidation is required for the respiration and proliferation of malignant glioma cells. Neuro Oncol.

[11] Wang S (2024). Lactate reprograms glioblastoma immunity through CBX3-regulated histone lactylation. J Clin Invest.

[12] Lv D (2024). Metabolic regulation of the glioblastoma stem cell epitranscriptome by malate dehydrogenase 2. Cell Metab.

[13] Galluzzi L (2013). Metabolic targets for cancer therapy. Nat Rev Drug Discov.

[14] Bezwada D (2024). Mitochondrial complex I promotes kidney cancer metastasis. Nature.

[15] Luengo A (2017). Targeting metabolism for cancer therapy. Cell Chem Biol.

[16] DeBerardinis RJ, Chandel NS (2016). Fundamentals of cancer metabolism. Sci Adv.

[17] Vander Heiden MG, DeBerardinis RJ (2017). Understanding the intersections between metabolism and cancer biology. Cell.

[18] Nguyen TTT (2020). HDAC inhibitors elicit metabolic reprogramming by targeting super-enhancers in glioblastoma models. J Clin Invest.

[19] Kumar PR (2022). PGC-1α induced mitochondrial biogenesis in stromal cells underpins mitochondrial transfer to melanoma. Br J Cancer.

[20] Chaudhary S (2021). PGC1A driven enhanced mitochondrial DNA copy number predicts outcome in pediatric acute myeloid leukemia. Mitochondrion.

[21] Valcarcel-Jimenez L (2019). PGC1α suppresses prostate cancer cell invasion through ERRα transcriptional control. Cancer Res.

[22] Kresovich JK (2018). Promoter methylation of PGC1A and PGC1B predicts cancer incidence in a veteran cohort. Epigenomics.

[23] Cai FF (2016). Prognostic value of plasma levels of HIF-1a and PGC-1a in breast cancer. Oncotarget.

[24] Carracedo A (2012). A metabolic prosurvival role for PML in breast cancer. J Clin Invest.

[25] Zuo X (2019). PPARD and interferon gamma promote transformation of gastric progenitor cells and tumorigenesis in mice. Gastroenterology.

[26] Gregory MA (2003). Phosphorylation by glycogen synthase kinase-3 controls c-myc proteolysis and subnuclear localization. J Biol Chem.

[27] Wang J (2008). c-Myc is required for maintenance of glioma cancer stem cells. PLoS One.

[28] Li F (2024). A peptide encoded by upstream open reading frame of *MYC* binds to tropomyosin receptor kinase B and promotes glioblastoma growth in mice. Sci Transl Med.

[29] Lin P (2024). RBBP6 maintains glioblastoma stem cells through CPSF3-dependent alternative polyadenylation. Cell Discov.

[30] Li J (2021). PI3Kγ inhibition suppresses microglia/TAM accumulation in glioblastoma microenvironment to promote exceptional temozolomide response. Proc Natl Acad Sci U S A.

[31] Haddad TC (2023). Evaluation of alisertib alone or combined with fulvestrant in patients with endocrine-resistant advanced breast cancer: The phase 2 TBCRC041 randomized clinical trial. JAMA Oncol.

[32] Shang E (2020). Epigenetic targeting of Mcl-1 is synthetically lethal with Bcl-xL/Bcl-2 inhibition in model systems of glioblastoma. Cancers (Basel).

[33] Meng W (2018). CDK7 inhibition is a novel therapeutic strategy against GBM both in vitro and in vivo. Cancer Manag Res.

[34] Yao Y (2023). CDK7 controls E2F- and MYC-driven proliferative and metabolic vulnerabilities in multiple myeloma. Blood.

[35] Funke K (2022). Transcriptional CDK inhibitors as potential treatment option for testicular germ cell tumors. Cancers (Basel).

[36] Zhang H (2020). CDK7 inhibition potentiates genome instability triggering anti-tumor immunity in small cell lung cancer. Cancer Cell.

[37] Olson CM (2019). Development of a selective CDK7 covalent inhibitor reveals predominant cell-cycle phenotype. Cell Chem Biol.

[38] Dai W (2022). CDK12 orchestrates super-enhancer-associated CCDC137 transcription to direct hepatic metastasis in colorectal cancer. Clin Transl Med.

[39] Emadi F (2020). CDK12: a potential therapeutic target in cancer. Drug Discov Today.

[40] Liu S (2023). Targeting CDK12 obviates the malignant phenotypes of colorectal cancer through the Wnt/β-catenin signaling pathway. Exp Cell Res.

[41] Quereda V (2019). Therapeutic targeting of CDK12/CDK13 in triple-negative breast cancer. Cancer Cell.

[42] Zhang T (2016). Covalent targeting of remote cysteine residues to develop CDK12 and CDK13 inhibitors. Nat Chem Biol.

[43] Gomez-Bougie P (2011). Noxa controls Mule-dependent Mcl-1 ubiquitination through the regulation of the Mcl-1/USP9X interaction. Biochem Biophys Res Commun.

[44] Liu M (2022). The mechanism of BUD13 m6A methylation mediated MBNL1-phosphorylation by CDK12 regulating the vasculogenic mimicry in glioblastoma cells. Cell Death Dis.

[45] Reardon DA (2016). Glioblastoma eradication following immune checkpoint blockade in an orthotopic, immunocompetent model. Cancer Immunol Res.

[46] Kim KS (2024). Fc-enhanced anti-CTLA-4, anti-PD-1, doxorubicin, and ultrasound-mediated blood-brain barrier opening: A novel combinatorial immunotherapy regimen for gliomas. Neuro Oncol.

[47] Zamler DB (2022). Immune landscape of a genetically engineered murine model of glioma compared with human glioma. JCI Insight.

[48] Haddad AF (2021). Mouse models of glioblastoma for the evaluation of novel therapeutic strategies. Neurooncol Adv.

[49] Grzelak CA (2022). Elimination of fluorescent protein immunogenicity permits modeling of metastasis in immune-competent settings. Cancer Cell.

[50] Day CP (2014). “Glowing head” mice: a genetic tool enabling reliable preclinical image-based evaluation of cancers in immunocompetent allografts. PLoS One.

[51] Fendt SM (2013). Reductive glutamine metabolism is a function of the α-ketoglutarate to citrate ratio in cells. Nat Commun.

[52] Metallo CM (2011). Reductive glutamine metabolism by IDH1 mediates lipogenesis under hypoxia. Nature.

[53] Filippone MG (2022). CDK12 promotes tumorigenesis but induces vulnerability to therapies inhibiting folate one-carbon metabolism in breast cancer. Nat Commun.

[54] Ishida CT (2018). Metabolic reprogramming by dual AKT/ERK inhibition through imipridones elicits unique vulnerabilities in glioblastoma. Clin Cancer Res.

[55] Hou X (2018). CDK6 inhibits white to beige fat transition by suppressing RUNX1. Nat Commun.

[56] Bayik D (2020). Myeloid-derived suppressor cell subsets drive glioblastoma growth in a sex-specific manner. Cancer Discov.

[57] Dagher OK (2023). Forks in the road for CAR T and CAR NK cell cancer therapies. Nat Immunol.

[58] Palmer AC (2022). Predictable clinical benefits without evidence of synergy in trials of combination therapies with immune-checkpoint inhibitors. Clin Cancer Res.

[59] Jerby-Arnon L (2018). A cancer cell program promotes T cell exclusion and resistance to checkpoint blockade. Cell.

[60] Pang L (2023). Hypoxia-driven protease legumain promotes immunosuppression in glioblastoma. Cell Rep Med.

[61] Pant A, Lim M (2023). CAR-T therapy in GBM: current challenges and avenues for improvement. Cancers (Basel).

[62] Antonarakis ES (2020). *CDK12*-altered prostate cancer: clinical features and therapeutic outcomes to standard systemic therapies, poly (ADP-ribose) polymerase inhibitors, and PD-1 inhibitors. JCO Precis Oncol.

[63] Wu Z (2024). CDK12 inhibition upregulates ATG7 triggering autophagy via AKT/FOXO3 pathway and enhances anti-PD-1 efficacy in colorectal cancer. Pharmacol Res.

[64] Li Y (2020). CDK12/13 inhibition induces immunogenic cell death and enhances anti-PD-1 anticancer activity in breast cancer. Cancer Lett.

