# Supplementary material for: CDK12 regulates cellular metabolism to promote glioblastoma growth
Source: JCI Insight. 2025 Sep 25;10(21):e190780. doi: 10.1172/jci.insight.190780 (PMC12643520; doi:10.1172/jci.insight.190780)
Supplement: Supplemental data [file jciinsight-10-190780-s184.pdf]

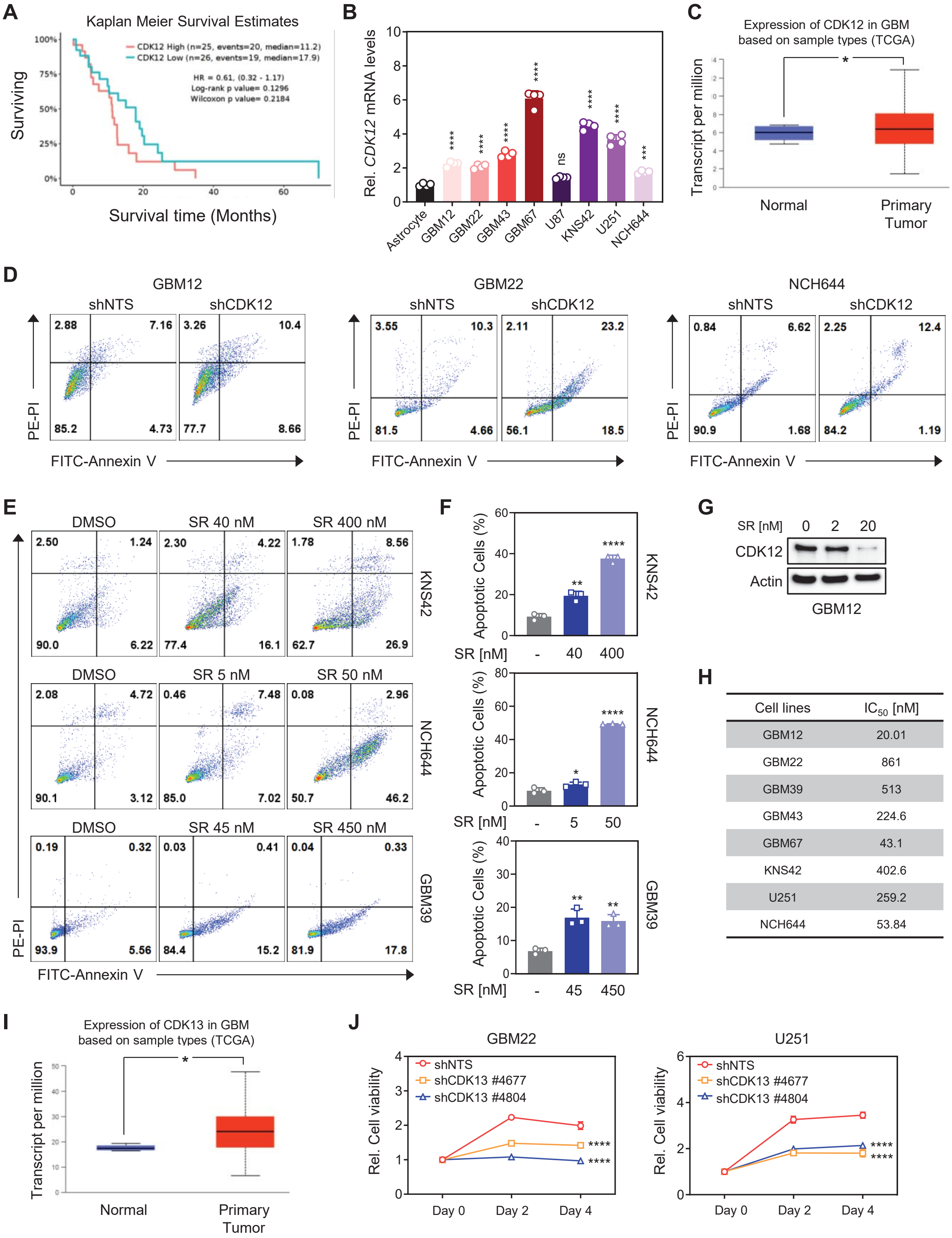
A
Kaplan Meier Survival Estimates
CDK12 High (n=25, events=20, median=11.2)
CDK12 Low (n=26, events=19, median=17.9)
HR = 0.61, (0.32 - 1.17)
Log-rank p value= 0.1296
Wilcoxon p value= 0.2184
Surviving
Survival time (Months)
B
Rel. CDK12 mRNA levels
Astrocyte
GBM12
GBM22
GBM43
GBM67
U87
KNS42
U251
NCH644
ns
C
Expression of CDK12 in GBM based on sample types (TCGA)
Transcript per million
Normal
Primary Tumor
D
GBM12
GBM22
NCH644
shNTS
shCDK12
PE-PI
FITC-Annexin V
2.88
7.16
85.2
4.73
3.26
10.4
77.7
8.66
3.55
10.3
81.5
4.66
2.11
23.2
56.1
18.5
0.84
6.62
90.9
1.68
2.25
12.4
84.2
1.19
E
DMSO
SR 40 nM
SR 400 nM
2.50
1.24
90.0
6.22
2.30
4.22
77.4
16.1
1.78
8.56
62.7
26.9
KNS42
DMSO
SR 5 nM
SR 50 nM
2.08
4.72
90.1
3.12
0.46
7.48
85.0
7.02
0.08
2.96
50.7
46.2
NCH644
DMSO
SR 45 nM
SR 450 nM
0.19
0.32
93.9
5.56
0.03
0.41
84.4
15.2
0.04
0.33
81.9
17.8
GBM39
PE-PI
FITC-Annexin V
F
Apoptotic Cells (%)
SR [nM]
-
40
400
KNS42
5
50
NCH644
45
450
GBM39
G
SR [nM]
0
2
20
CDK12
Actin
GBM12
H
Cell lines
IC50 [nM]
GBM12 20.01
GBM22 861
GBM39 513
GBM43 224.6
GBM67 43.1
KNS42 402.6
U251 259.2
NCH644 53.84
I
Expression of CDK13 in GBM based on sample types (TCGA)
Transcript per million
Normal
Primary Tumor
J
GBM22
U251
shNTS
shCDK13 #4677
shCDK13 #4804
Rel. Cell viability
Day 0
Day 2
Day 4

Supplemental Figure 1. CDK12 is a therapeutic target in GBM. (**A**) Kaplan-Meier curve in GBM TCGA cohort (TCGA_GBM, RNA-SEQ, CDK12, GBM, Mesenchymal, Gender: all, IDH: All, Recurrence: all, MGMT: all) comparing survival rates of GBM patient samples divided into high-CDK12 and low-CDK12 based on CDK12 mRNA expression (HR = 0.61, 95% CI: 0.32–1.17; log-rank *P* = 0.1296; Wilcoxon *P* = 0.2184). High-CDK12 levels correlate with a worse overall survival in GBM patients. (**B**) CDK12 mRNA levels were quantified using qPCR and compared between astrocytes and multiple GBM cells. Data are presented as mean ± SD. ***p < 0.001; ****p < 0.0001; ns. not significant, one-way ANOVA with Dunnett's multiple comparisons test. (**C**) CDK12 expression validation in TCGA database on GBM samples by UALCAN (https://ualcan.path.uab.edu/cgi-bin/ualcan-res-cbttc.pl). (**D**) GBM12, GBM22, and NCH644 cells expressing shNTS or shCDK12 cells were analyzed by flow cytometry following staining with Annexin V/PI. (**E-F**) KNS42, NCH644, and GBM39 cells were treated with SR for 72 h and were analyzed by flow cytometry with Annexin V/PI staining. Bar graph shows percentage of apoptosis cells. Data are presented as mean ± SD. *p < 0.05; **p < 0.01; ****p < 0.0001, one-way ANOVA with Dunnett's multiple comparisons test. (**G**) Western blot shows CDK12 protein levels after SR treatment in GBM12 cells. Actin was used as a loading control. (**H**) Multiple GBM cells; GBM12, GBM22, GBM39, GBM43, GBM67, KNS42, U251, and NCH644 were treated with SR for 72 h, then performed to cell viability assays. There are listed IC_{50} values, respectively. (**I**) CDK13 expression validation in TCGA database on GBM samples by UALCAN. (**J**) Stable shCDK13 cells (no. 4677, 4804), or shNTS cells were assessed for cell viability for 0, 2, and 4 days. Data are presented as mean ± SD. ****p < 0.0001, one-way ANOVA with Dunnett's multiple comparisons test.

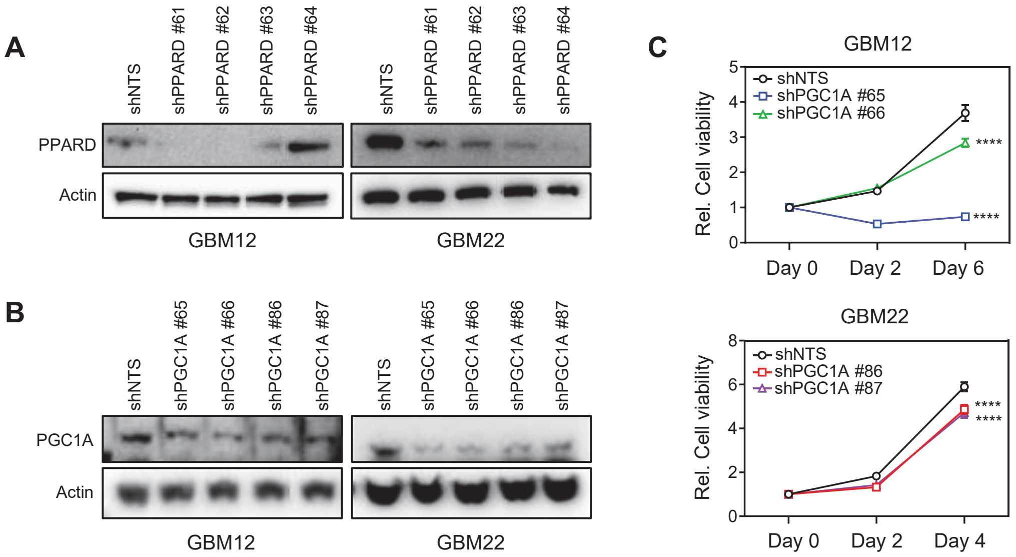
A
shNTS
shPPARD #61
shPPARD #62
shPPARD #63
shPPARD #64
PPARD
Actin
GBM12
GBM22
B
shNTS
shPGC1A #65
shPGC1A #66
shPGC1A #86
shPGC1A #87
PGC1A
Actin
GBM12
GBM22
C
GBM12
Rel. Cell viability
shNTS
shPGC1A #65
shPGC1A #66
****
****
Day 0
Day 2
Day 6
GBM22
shNTS
shPGC1A #86
shPGC1A #87
****
****
Day 0
Day 2
Day 4

Supplemental Figure 2. PGC1A is a driver of proliferation in PDX GBM cultures. (**A**) Western blot shows PPARD protein levels in shPPARD (no. 61,62,63,64) cells compared to shNTS cells in GBM12, and GBM22 cells. (**B**) Western blot shows in PGC1A protein levels in shPGC1A (no. 65,66,86,87) cells compared to shNTS cells in GBM12, and GBM22 cells. (**C**) Cell viability of GBM12, GBM22-shPGC1A cells compared to shNTS cells was assessed for 0, 2, 4, or 6 days. Data are presented as mean ± SD. ****$p < 0.0001$, one-way ANOVA with Dunnett's multiple comparisons test.

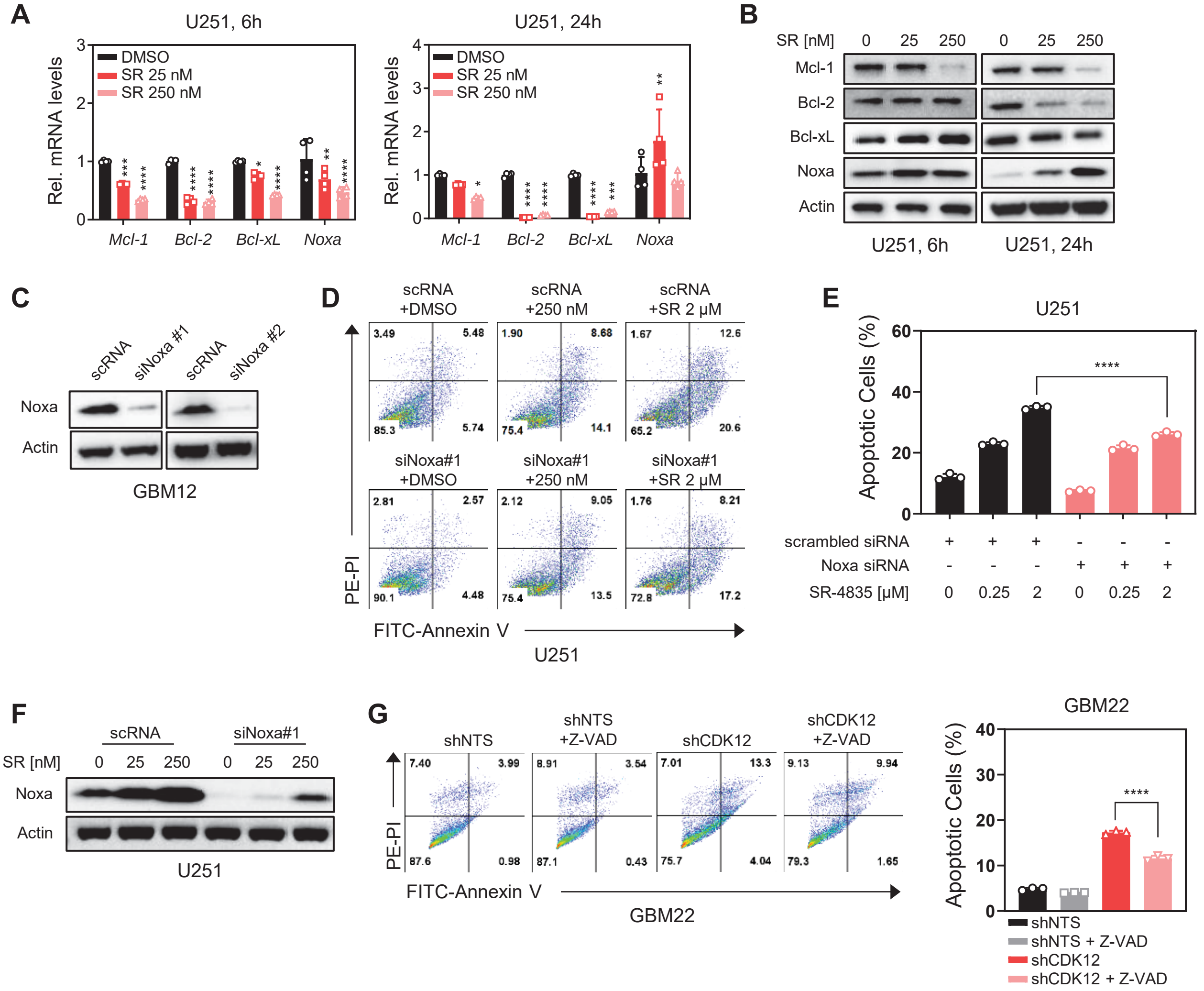
A
U251, 6h
U251, 24h
Rel. mRNA levels
DMSO
SR 25 nM
SR 250 nM
Mcl-1
Bcl-2
Bcl-xL
Noxa
B
SR [nM]
0
25
250
Mcl-1
Bcl-2
Bcl-xL
Noxa
Actin
U251, 6h
U251, 24h
C
scRNA
siNoxa #1
siNoxa #2
Noxa
Actin
GBM12
D
scRNA +DMSO
scRNA +250 nM
scRNA +SR 2 μM
siNoxa#1 +DMSO
siNoxa#1 +250 nM
siNoxa#1 +SR 2 μM
PE-PI
FITC-Annexin V
U251
E
U251
Apoptotic Cells (%)
scrambled siRNA
Noxa siRNA
SR-4835 [μM]
F
scRNA
siNoxa#1
SR [nM]
Noxa
Actin
U251
G
shNTS
shNTS +Z-VAD
shCDK12
shCDK12 +Z-VAD
PE-PI
FITC-Annexin V
GBM22
Apoptotic Cells (%)
shNTS
shNTS + Z-VAD
shCDK12
shCDK12 + Z-VAD

Supplemental Figure 3. Noxa, a mediator of cell death, plays a crucial role in the response of GBM cells to the treatment of SR-4835. (**A**) The qPCR analysis in apoptosis related genes of U251 cells treated with increasing concentrations of SR for 6 h and 24 h. qPCR data are presented as mean ± SD. *$p < 0.05$; **$p < 0.01$; ***$p < 0.001$; ****$p < 0.0001$, one-way ANOVA with Dunnett's multiple comparisons test. (**B**) U251 cells were treated with SR for 6 h or 24 h cell lysates were subjected to standard western blotting with the indicated antibodies the anti-apoptotic Bcl-2 family members (Mcl-1, Bcl-xL, and Bcl-2) and pro-apoptotic protein (Noxa). (**C**) GBM12 cells were transfected with scRNA or siNoxa#1, 2, and detected by western blot with anti-Noxa. (**D-E**) U251 cells were transfected with siNoxa and treated with SR for 72 h and analyzed by flow cytometry using Annexin V/PI staining. Bar graph shows percentage of apoptotic cells. Data are presented as mean ± SD. ****$p < 0.0001$, one-way ANOVA with Dunnett's multiple comparisons test. (**F**) U251 cells were transfected with scRNA or siNoxa#1, and treated with SR, and detected by western blot with anti-Noxa. (**G**) GBM22-shNTS and GBM22-shCDK12 (1798) cells were treated with Z-VAD (50 μM). Apoptosis was assessed by flow cytometry using Annexin V/PI staining. The percentage of apoptotic cells was quantified. Data are presented as mean ± SD. ****$p < 0.0001$, one-way ANOVA with Dunnett's multiple comparisons test.

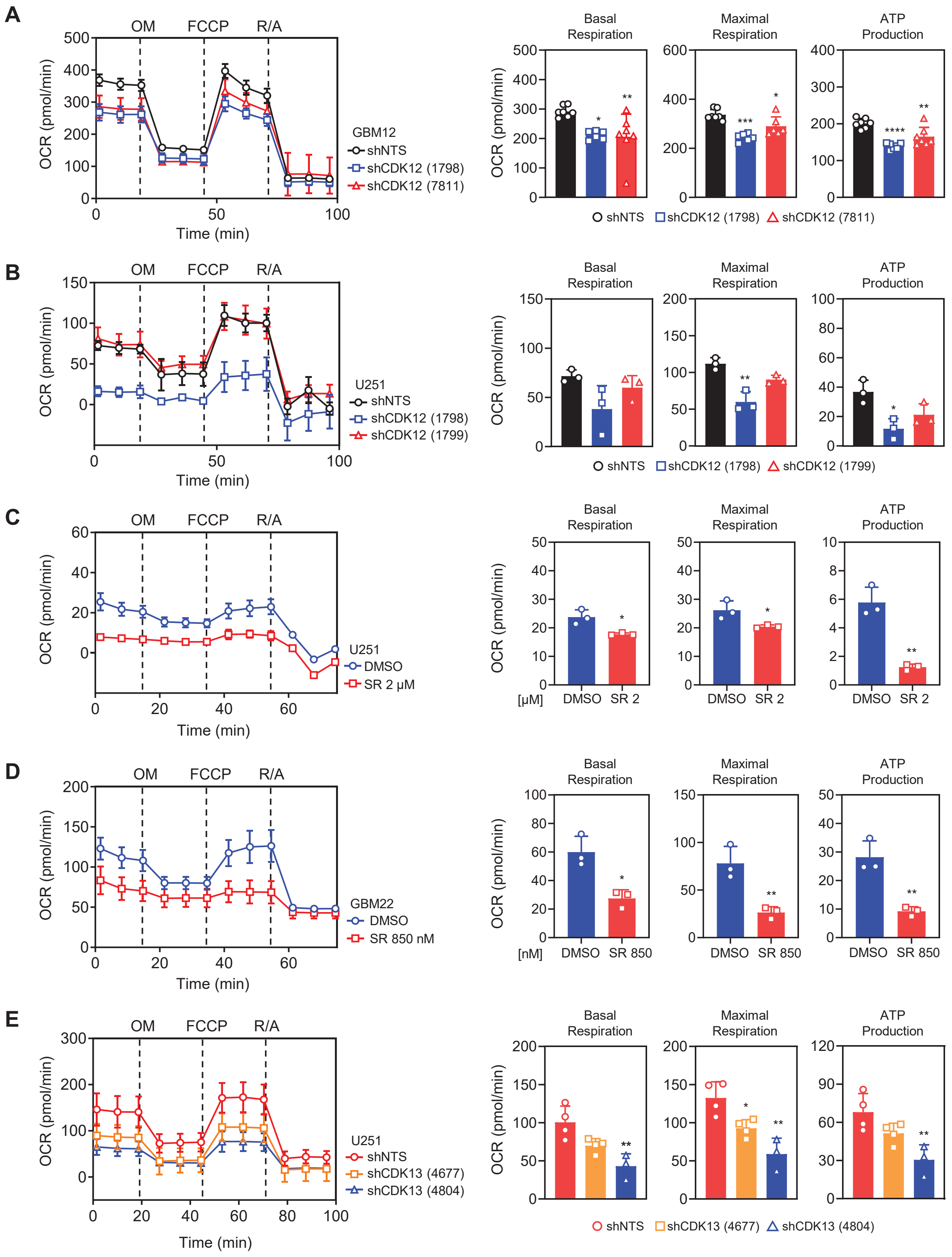

A
OM
FCCP
R/A
OCR (pmol/min)
Time (min)
GBM12
shNTS
shCDK12 (1798)
shCDK12 (7811)
Basal Respiration
Maximal Respiration
ATP Production
shNTS
shCDK12 (1798)
shCDK12 (7811)
B
U251
shNTS
shCDK12 (1798)
shCDK12 (1799)
C
U251
DMSO
SR 2 μM
[μM]
DMSO
SR 2
D
GBM22
DMSO
SR 850 nM
[nM]
DMSO
SR 850
E
U251
shNTS
shCDK13 (4677)
shCDK13 (4804)
shNTS
shCDK13 (4677)
shCDK13 (4804)

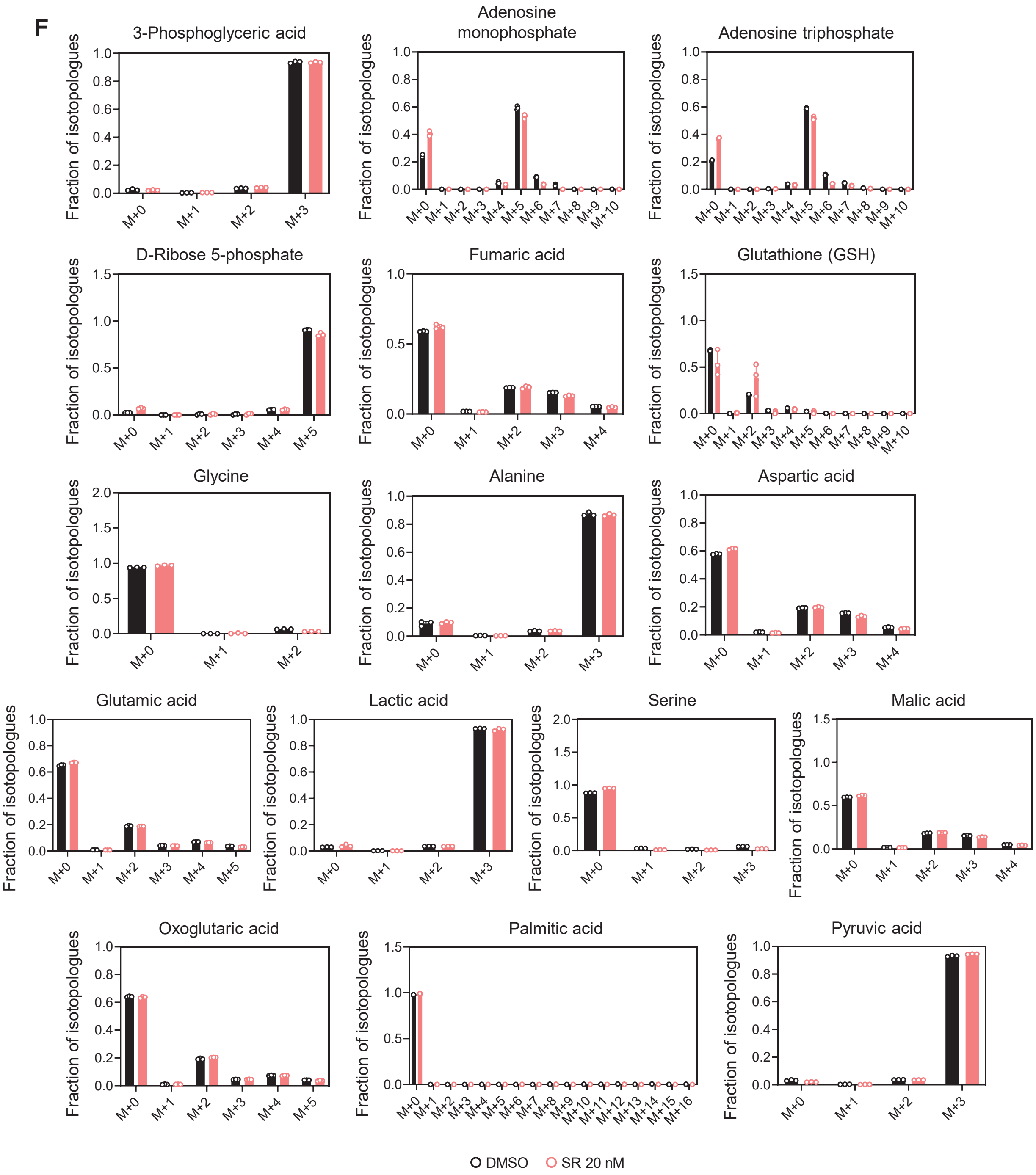

F
3-Phosphoglyceric acid
Adenosine monophosphate
Adenosine triphosphate
D-Ribose 5-phosphate
Fumaric acid
Glutathione (GSH)
Glycine
Alanine
Aspartic acid
Glutamic acid
Lactic acid
Serine
Malic acid
Oxoglutaric acid
Palmitic acid
Pyruvic acid
Fraction of isotopologues
DMSO
SR 20 nM

G

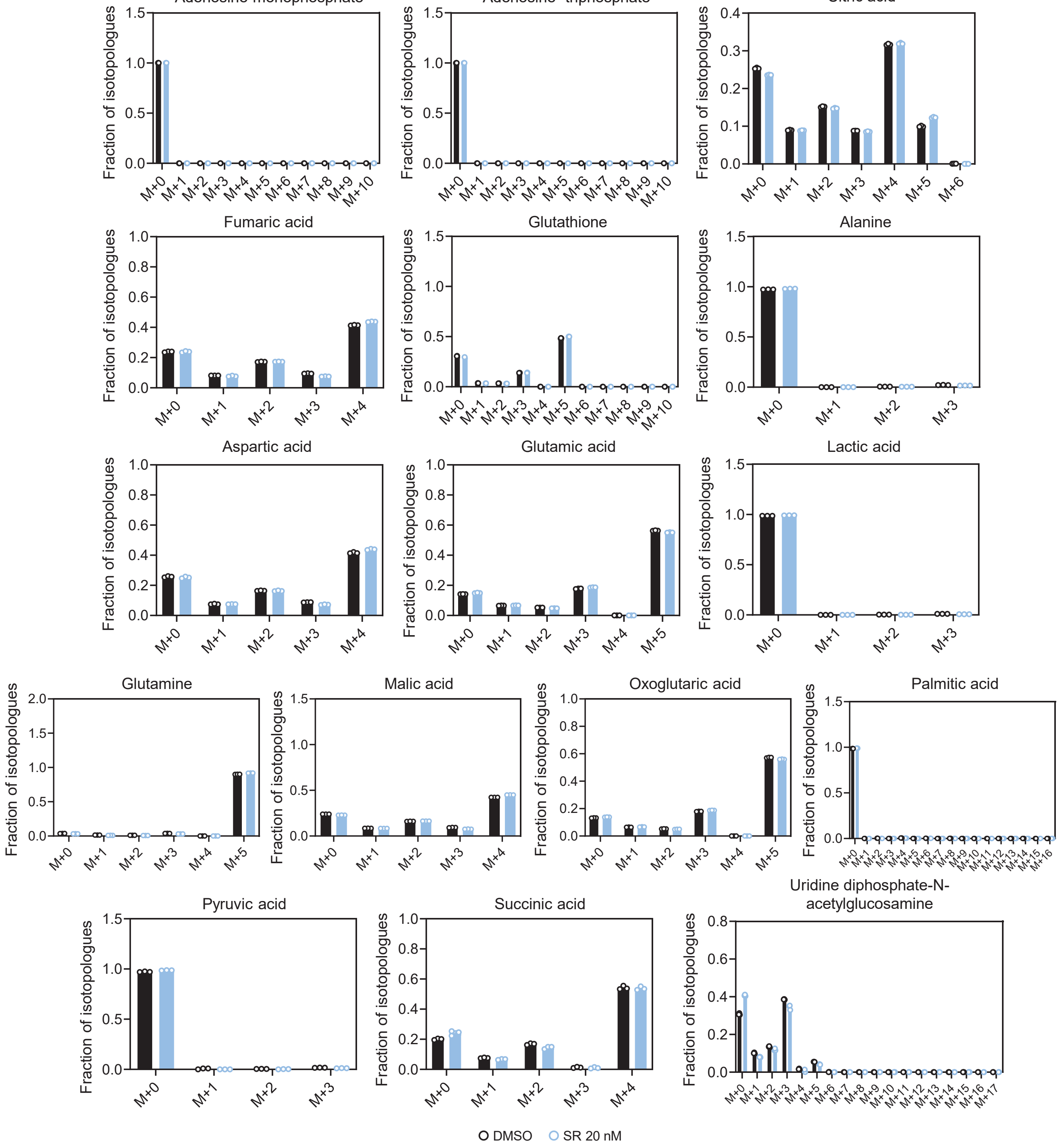

Adenosine monophosphate
Adenosine triphosphate
Citric acid
Fumaric acid
Glutathione
Alanine
Aspartic acid
Glutamic acid
Lactic acid
Glutamine
Malic acid
Oxoglutaric acid
Palmitic acid
Pyruvic acid
Succinic acid
Uridine diphosphate-N-acetylglucosamine
Fraction of isotopologues
M+0
M+1
M+2
M+3
M+4
M+5
M+6
M+7
M+8
M+9
M+10
M+11
M+12
M+13
M+14
M+15
M+16
M+17
DMSO
SR 20 nM

H

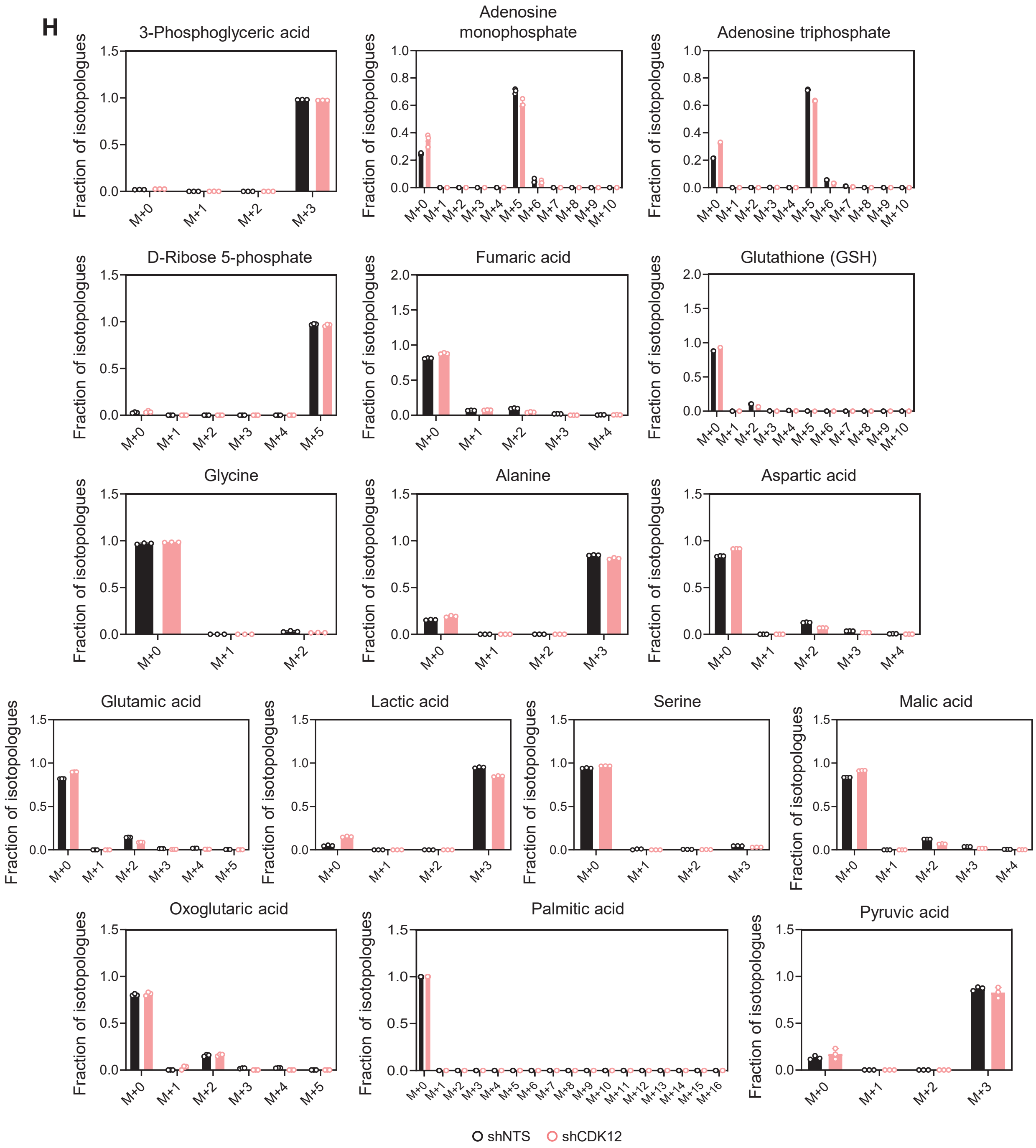
3-Phosphoglyceric acid
Adenosine monophosphate
Adenosine triphosphate
D-Ribose 5-phosphate
Fumaric acid
Glutathione (GSH)
Glycine
Alanine
Aspartic acid
Glutamic acid
Lactic acid
Serine
Malic acid
Oxoglutaric acid
Palmitic acid
Pyruvic acid
Fraction of isotopologues
M+0
M+1
M+2
M+3
M+4
M+5
M+6
M+7
M+8
M+9
M+10
M+11
M+12
M+13
M+14
M+15
M+16
shNTS
shCDK12

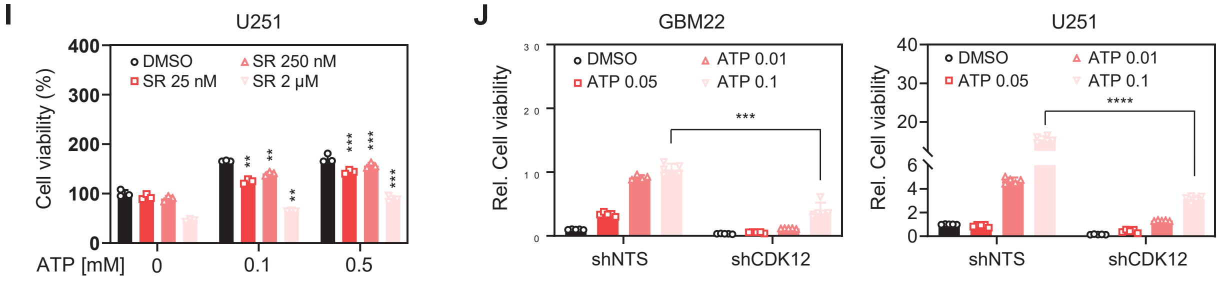

I
U251
Cell viability (%)
400
300
200
100
0
DMSO
SR 25 nM
SR 250 nM
SR 2 μM
**
**
**
***
***
***
ATP [mM]
0
0.1
0.5
J
GBM22
Rel. Cell viability
3 0
2 0
1 0
0
DMSO
ATP 0.05
ATP 0.01
ATP 0.1
***
shNTS
shCDK12
U251
Rel. Cell viability
40
30
20
6
4
2
0
DMSO
ATP 0.05
ATP 0.01
ATP 0.1
****
shNTS
shCDK12

Supplemental Figure 4. CDK12 regulates the oxygen consumption rate in GBM22 and U251 cells. (**A**) Seahorse mitochondrial stress assay was conducted on GBM12-shNTS, shCDK12 (1798, 7811) cells and (**B**) U251-shNTS, shCDK12 (1798, 1799) cells. Data are presented as mean ± SD. *p < 0.05; **p < 0.01; ***p < 0.001; ****p < 0.0001, one-way ANOVA with Dunnett's multiple comparisons test. (**C**) Seahorse mitochondrial stress assay of U251 cells treated DMSO or SR 2 μM for 24 h. Data are presented as mean ± SD. *p < 0.05; **p < 0.01, unpaired 2-tailed *t* test. (**D**) Seahorse mitochondrial stress assay of GBM22 cells treated DMSO or SR-4835 850 nM for 24 h. Data are presented as mean ± SD. *p < 0.05; **p < 0.01, unpaired 2-tailed *t* test. (**E**) Seahorse mitochondrial stress assay was conducted on U251-shNTS, shCDK13 (4677, 4804) cells. Data are presented as mean ± SD. *p < 0.05; **p < 0.01, one-way ANOVA with Dunnett's multiple comparisons test. (**F**) For glucose tracing, GBM12 cells were treated with SR 20 nM in DMEM containing 25 mM U-$^{13}C_6$-glucose, 4 mM glutamine, and 1.5% dialyzed FBS for 24 h. Shown are fractions of the isotopologues for each metabolite (n = 3, each group). (**G**) For glutamine tracing, GBM12 cells were treated with SR 20 nM in DMEM containing 25 mM glucose, 4 mmol/L U-$^{13}C_5$ Glutamine, and 1.5% dialyzed FBS for 24 h. Shown are fractions of the isotopologues for each metabolite (n = 3, each group). (**H**) For glucose tracing, GBM22-shNTS and shCDK12 (1798) cell lines conducted glucose tracing in DMEM containing 25 mM U-$^{13}C_6$-glucose, 4 mM glutamine, and 1.5% dialyzed FBS for 24 h. Shown are fractions of the isotopologues for each metabolite (n = 3, each group). (**I**) U251 cells were treated with SR at various concentrations and were evaluated for proliferation in the presence or absence of ATP for a 48-hour period. Data are presented as mean ± SD. **p < 0.01; ***p < 0.001, two-way ANOVA with Turkey's multiple comparisons test. (**J**) GBM22-shNTS and shCDK12 (1798) cells were evaluated for proliferation in the presence or absence of ATP for a 48-hour period. Loss of CDK12 in GBM22 or U251 cells was treated with ATP at various concentrations. Data are presented as mean ± SD. ***p < 0.001; ****p < 0.0001, two-way ANOVA with Turkey's multiple comparisons test.

A

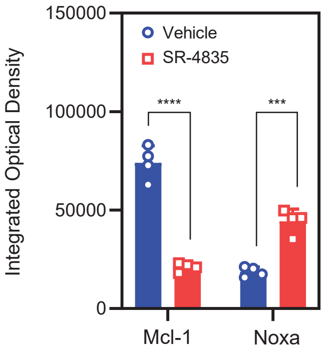

Integrated Optical Density
150000
100000
50000
0
Vehicle
SR-4835
****
***
Mcl-1
Noxa

Supplemental Figure 5. Immunohistochemical quantification of Mcl-1 and Noxa following SR-4835 treatment. (**A**) Quantification of IHC staining intensity with Mcl-1, and Noxa. A presented as a bar graph. Data are presented as mean ± SD. ***p < 0.001; ****p < 0.0001, unpaired two-tailed *t* test.

Supplemental Table 1. Primer sequences for qPCR.

**Gene list**	**5’ -> 3’**
qPCR primer: *18S* forward	ACCCGTTGAACCCCATTCGTGA
qPCR primer: *18S* reverse	GCCTCACTAAACCATCCAATCGG
qPCR primer: *CDK12* forward	CGAACTCAGCAAAATGGCTCCTC
qPCR primer: *CDK12* reverse	TTGGATGGAGGTGGCTCTTCGA
qPCR primer: *CPT1A* forward	GATCCTGGACAATACCTCGGAG
qPCR primer: *CPT1A* reverse	CTCCACAGCATCAAGAGACTGC
qPCR primer: *PGC1A* forward	CCAAAGGATGCGCTCTCGTTCA
qPCR primer: *PGC1A* reverse	CGGTGTCTGTAGTGGCTTGACT
qPCR primer: *PPARD* forward	GGCTTCCACTACGGTGTTCATG
qPCR primer: *PPARD* reverse	CTGGCACTTGTTGCGGTTCTTC
qPCR primer: *ACADSB* forward	GCCACCTATTTGCCTCAGCTCA
qPCR primer: *ACADSB* reverse	GCTCAGCACTGCTGATCCACAT
qPCR primer: *ACADVL* forward	TAGGAGAGGCAGGCAAACAGCT
qPCR primer: *ACADVL* reverse	CACAGTGGCAAACTGCTCCAGA
qPCR primer: *CPT2* forward	GCAGATGATGGTTGAGTGCTCC
qPCR primer: *CPT2* reverse	AGATGCCGCAGAGCAAACAAGTG
qPCR primer: *ETFDH* forward	GGAAACACCATCCTAGCATTCGG
qPCR primer: *ETFDH* reverse	CCACCAGGAAAGGTGAGTTTTGG
qPCR primer: *HADHB* forward	CACAGTCTAGCCAAGAAGGCAC
qPCR primer: *HADHB* reverse	CATCTGCTCCAGTGAGGAAGGA
qPCR primer: *Mcl-1* forward	CCAAGAAAGCTGCATCGAACCAT
qPCR primer: *Mcl-1* reverse	CAGCACATTCCTGATGCCACCT
qPCR primer: *Bcl-2* forward	ATCGCCCTGTGGATGACTGAGT
qPCR primer: *Bcl-2* reverse	GCCAGGAGAAATCAAACAGAGGC
qPCR primer: *Bcl-xL* forward	GCCACTTACCTGAATGACCACC
qPCR primer: *Bcl-xL* reverse	AACCAGCGGTTGAAGCGTTCCT
qPCR primer: *Noxa* forward	CTGGAAGTCGAGTGTGCTACTC
qPCR primer: *Noxa* reverse	TGAAGGAGTCCCCTCATGCAAG
