# Supplementary material for: CDK12 regulates cellular metabolism to promote glioblastoma growth
Source: JCI Insight. 2025 Sep 25;10(21):e190780. doi: 10.1172/jci.insight.190780 (PMC12643520; doi:10.1172/jci.insight.190780)

Uncropped WB images

Figure 1B

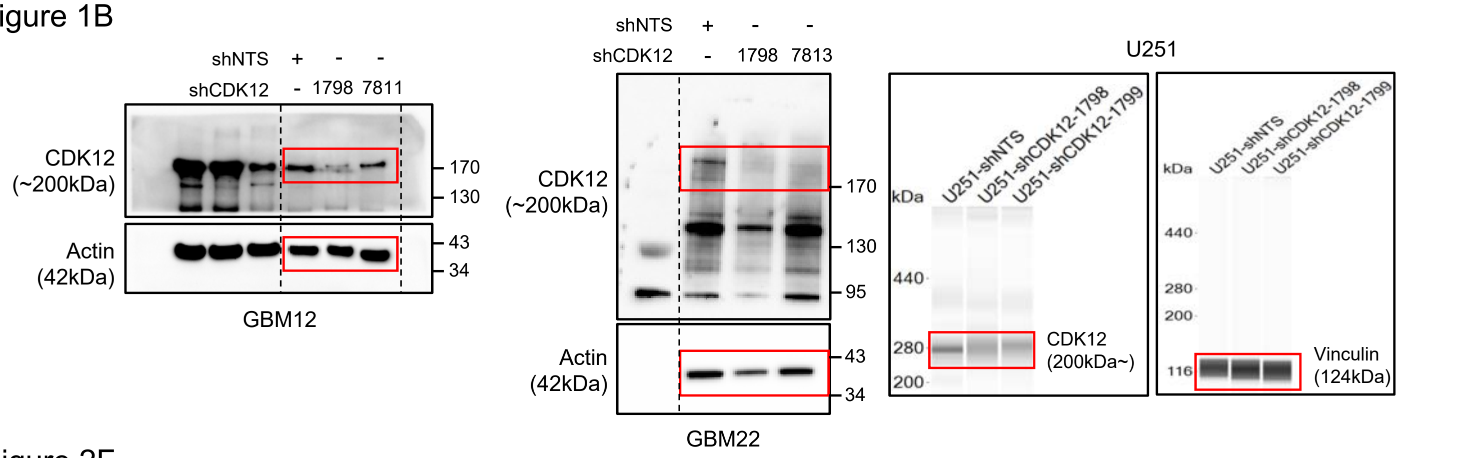

Figure 2F

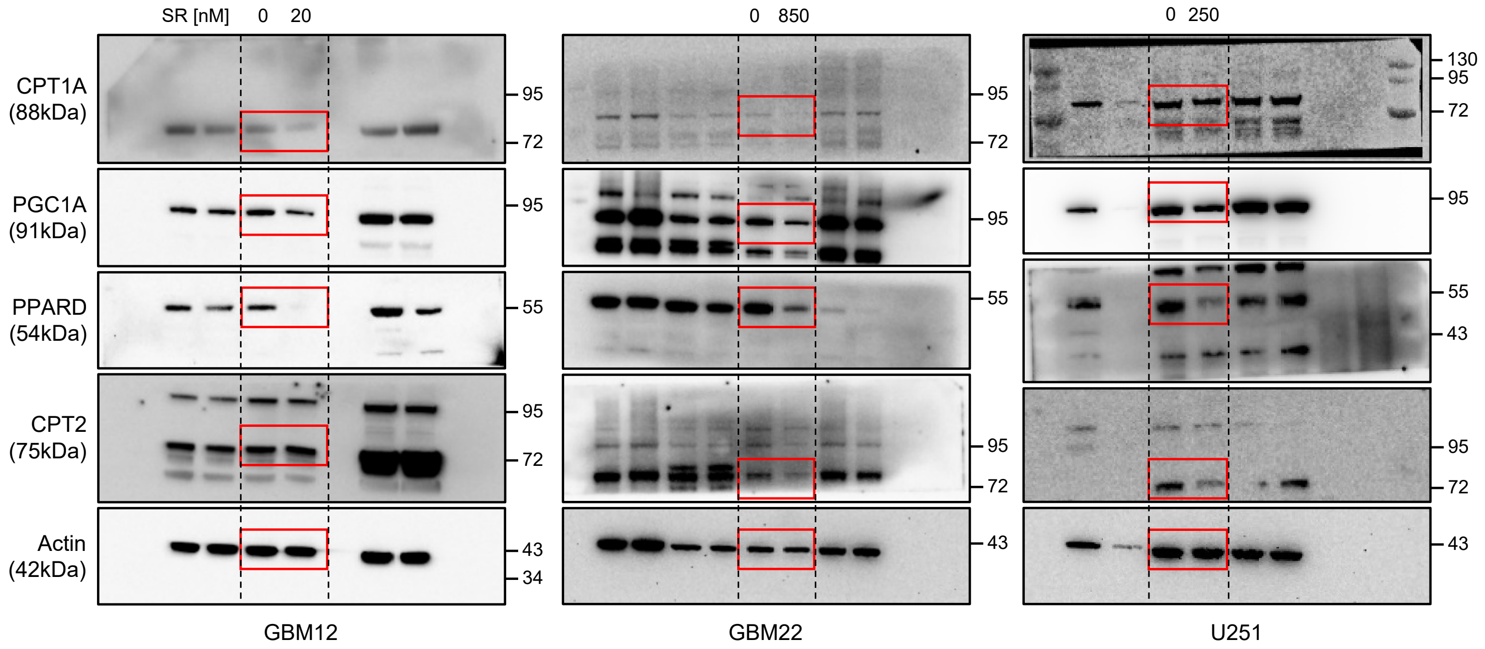

Figure 2G

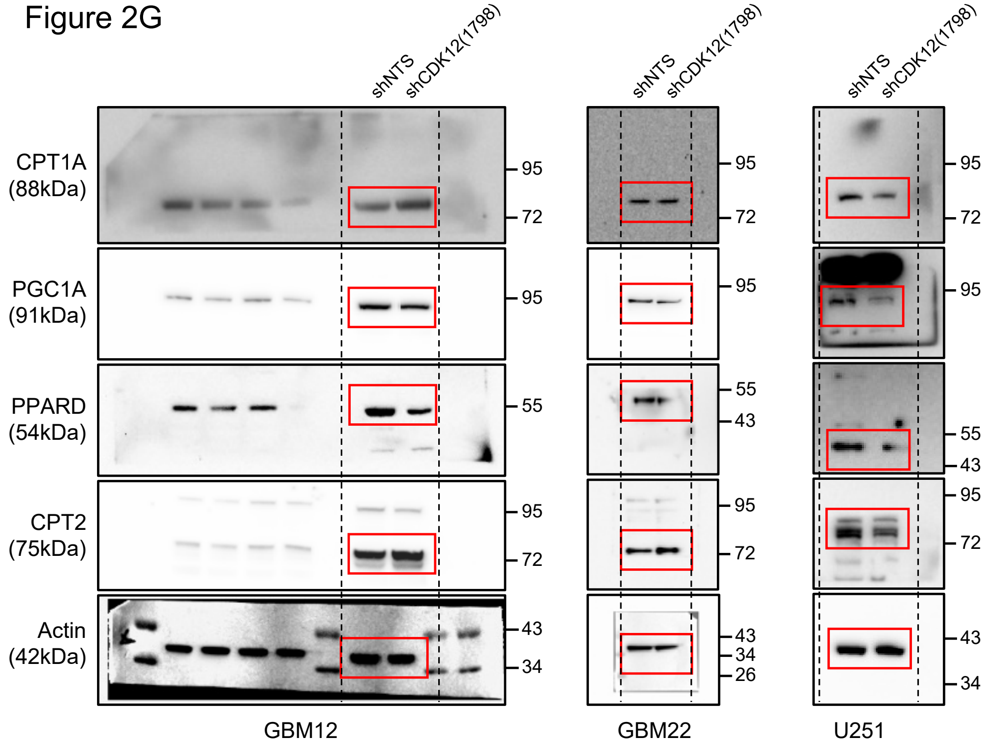

Figure 3E

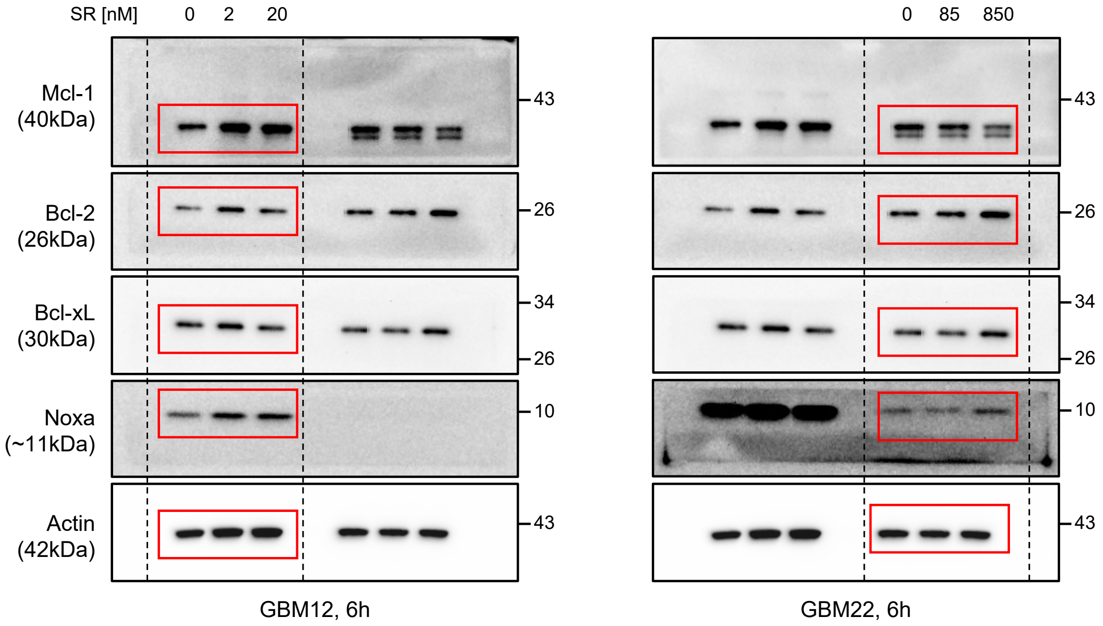

Figure 3F

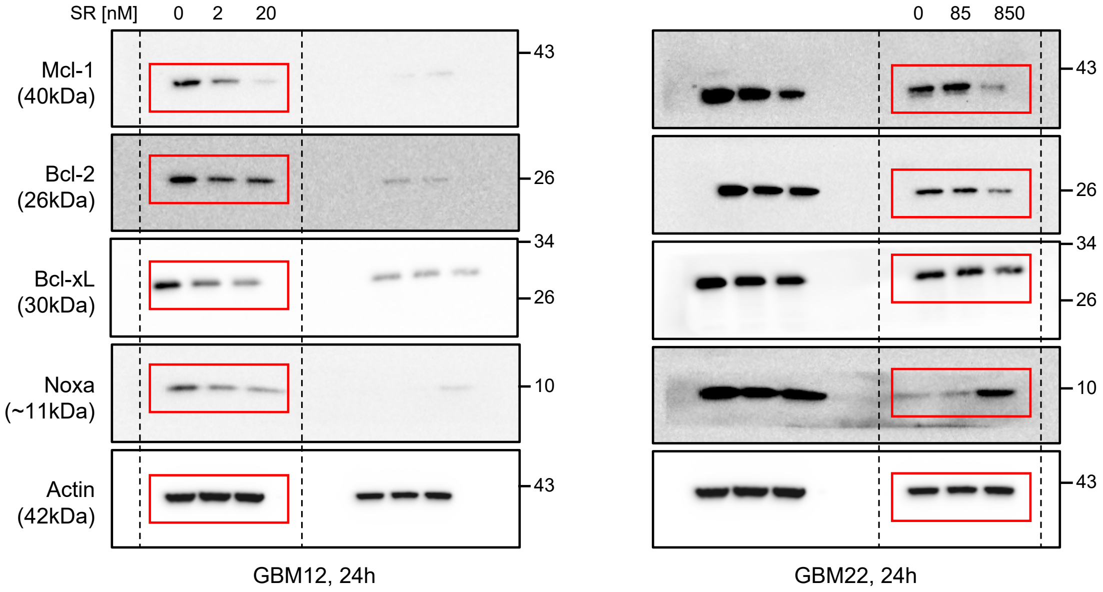

Figure 3G

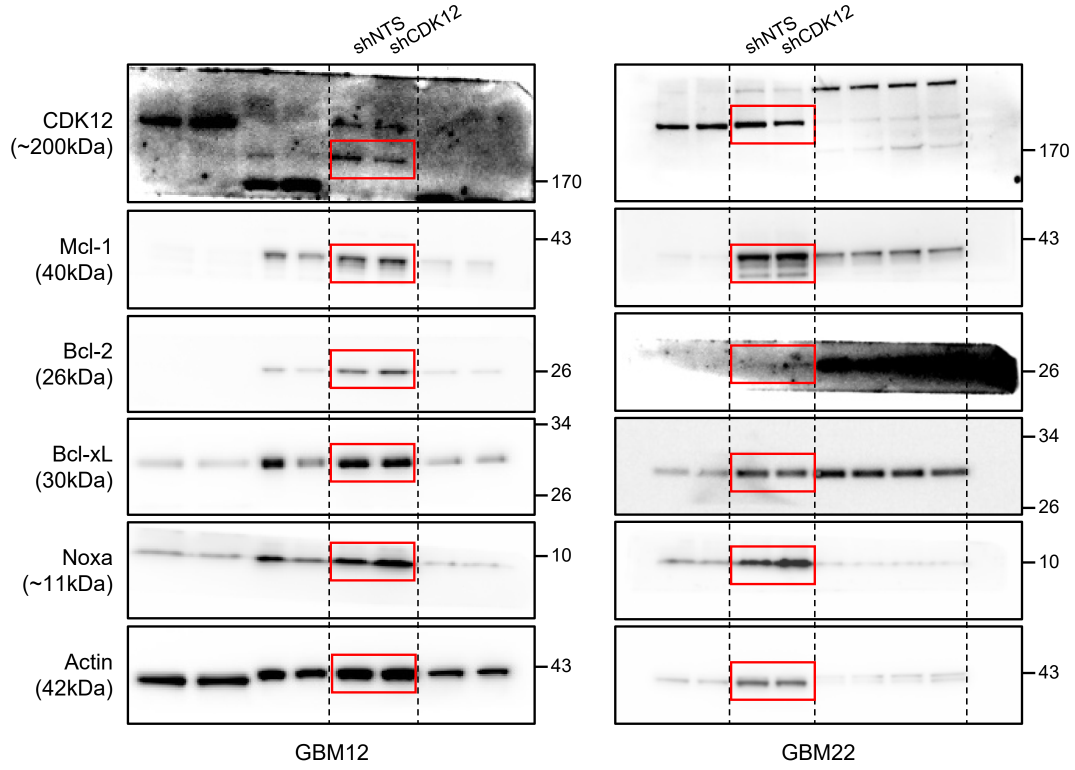

Figure 3H

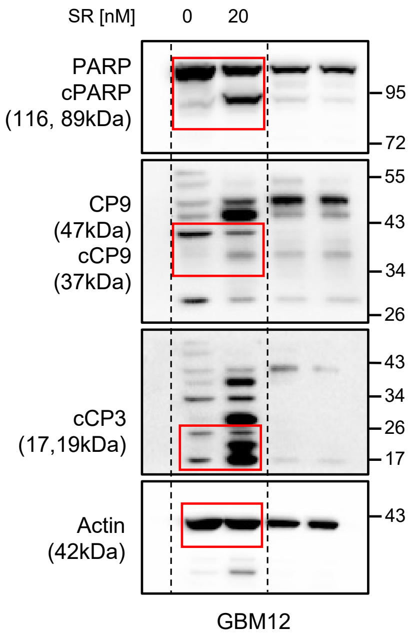

Figure 5A

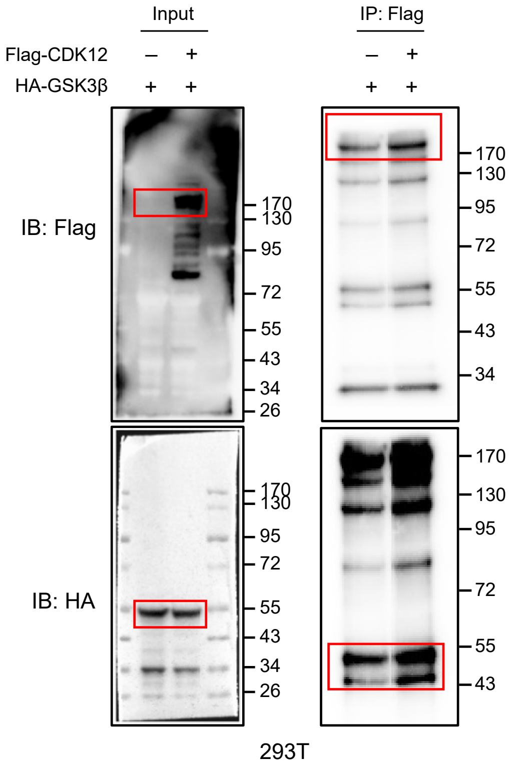

Figure 5B

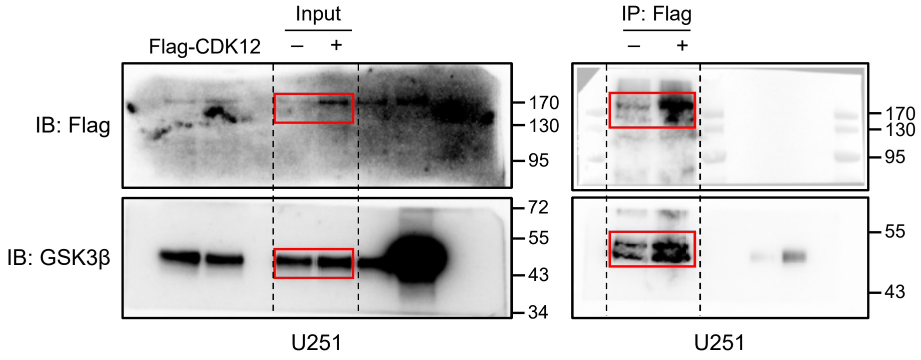

Figure 5C

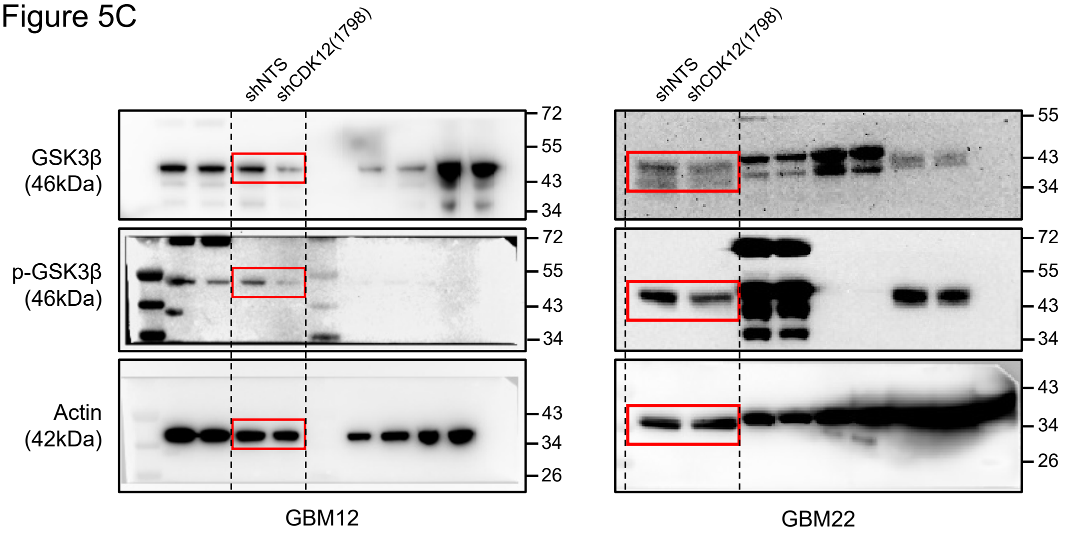

Figure 5D

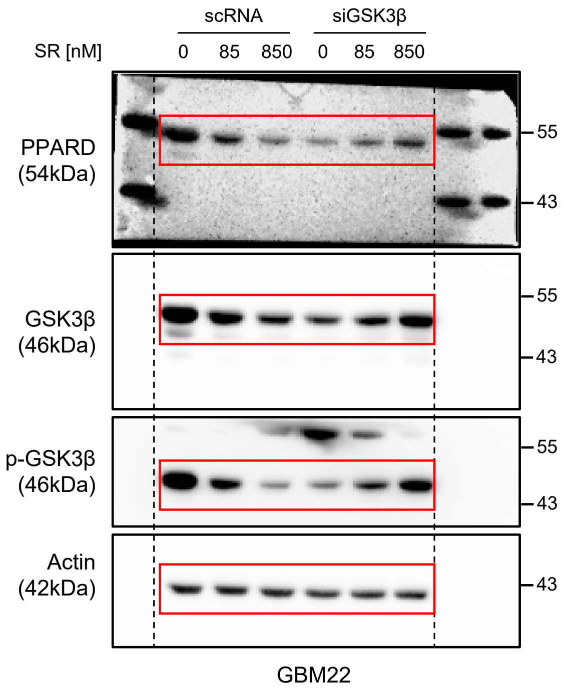

Figure 5E

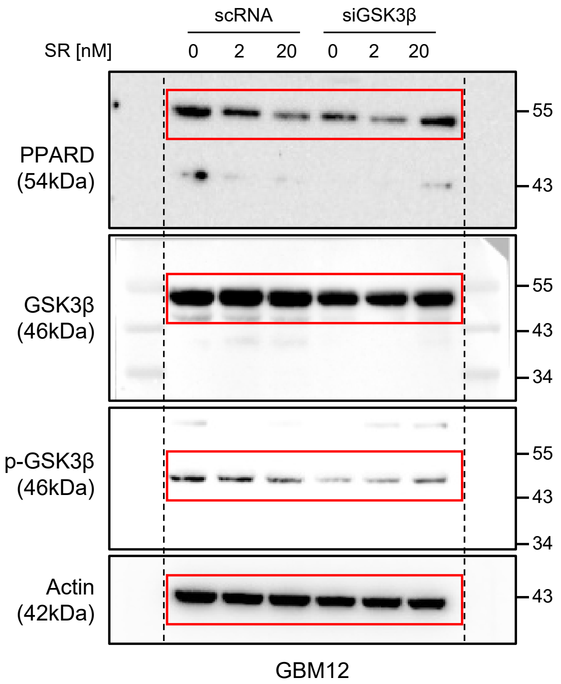

Supplementary Figure 1G

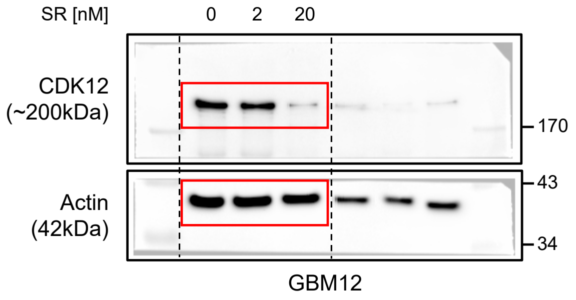

Supplementary Figure 2B

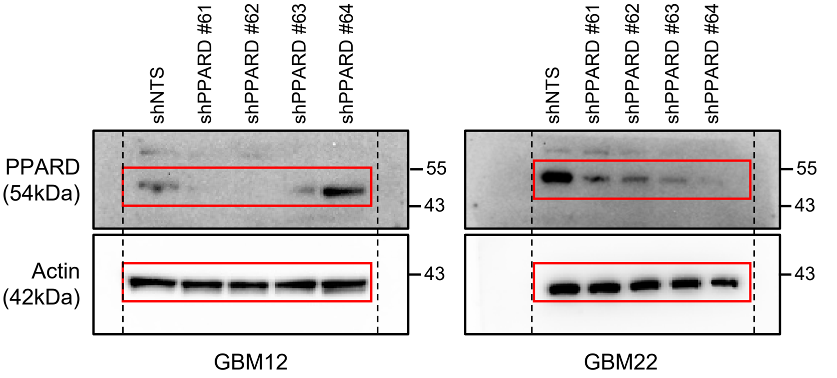

Supplementary Figure 2C

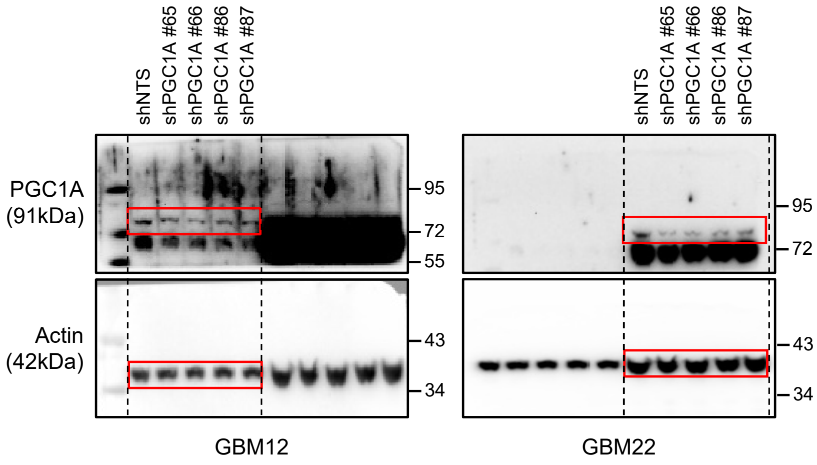

Supplementary Figure 3C

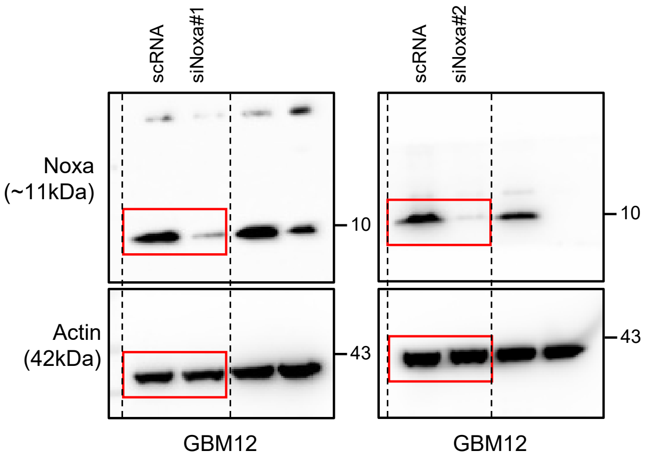

Supplementary Figure 3B

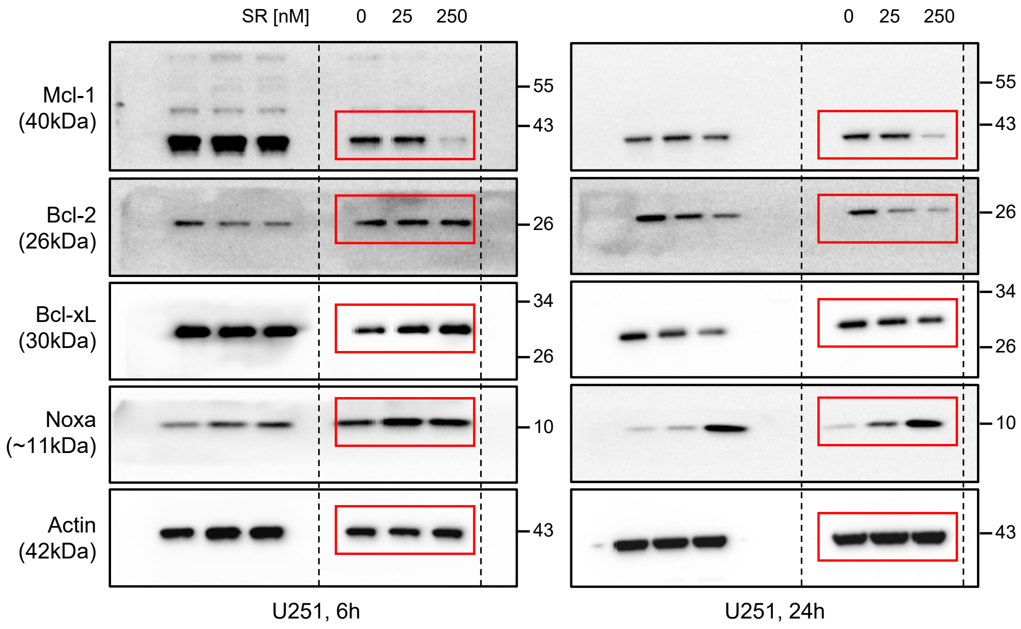

Supplementary Figure 3F

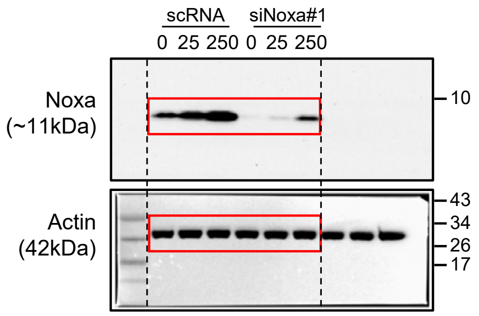

Supplement: Unedited blot and gel images [file jciinsight-10-190780-s185.pdf]
